# Spatial Organization of Expanding Bacterial Colonies Is Affected by Contact-Dependent Growth Inhibition

**DOI:** 10.1016/j.cub.2019.08.074

**Published:** 2019-11-04

**Authors:** Michael J. Bottery, Ioannis Passaris, Calvin Dytham, A. Jamie Wood, Marjan W. van der Woude

**Affiliations:** 1 Centre for Immunology and Infection and Hull York Medical School, University of York, York YO10 5DD, UK; 2 Department of Biology, University of York, York YO10 5DD, UK; 3 Department of Mathematics, University of York, York YO10 5DD, UK; 4 York Biomedical Research Institute, University of York YO10 5DD, UK

**Keywords:** contact-dependent inhibition, Escherichia coli, spatial structure, competition, CDI, individual-based modeling, competition systems, type V secretion system

## Abstract

Identifying how microbes are able to manipulate, survive, and thrive in complex multispecies communities has expanded our understanding of how microbial ecosystems impact human health and the environment. The ability of bacteria to negatively affect neighbors, through explicit toxin delivery systems, provides them with an opportunity to manipulate the composition of growing microbial communities. Contact-dependent inhibition (CDI) systems (a Type Vb secretion system) are a distinct subset of competition systems whose contribution to shaping the development of spatially structured bacterial communities are yet to be fully understood. Here, we compare the impact of different CDI systems, at both the single-cell and population level, to determine the key drivers of CDI-mediated competition within spatially structured bacterial populations. Through an iterative approach using both an *Escherichia coli* experimental system and computational modeling, we show that CDI systems have subtle and system-specific effects at the single-cell level, generating single-cell-wide boundaries between CDI-expressing inhibitor cells and their neighboring targets. Despite the subtle effects of CDI at a single-cell level, CDI systems greatly diminished the ability of susceptible targets to expand their range during colony growth. The inoculum density of the population, together with the CDI system-specific variables of the speed of inhibition after contact and biological cost of CDI, strongly affects CDI-mediated competition. In contrast, the magnitude of the toxin-induced growth retardation of target cells only weakly impacts the composition of the population. Our work reveals how distinct CDI systems can differentially affect the composition and spatial arrangement of bacterial populations.

## Highlights

• CDI causes subtle growth inhibition in a subset of contacted target cells
• Model describes and predicts observed effects on spatial distribution of strains
• CDI facilitates success of inhibitor strain increasing population patch size
• A CDI system’s inhibition rate dominates toxicity in driving competition outcome

## Teaser

Bottery et al. use an interdisciplinary approach to analyze contact-dependent inhibition in bacteria. CDI-dependent growth rate reduction is subtle but facilitates increased patch size for inhibitor cells in competitions on solid media. Of the variables impacting outcome, it is striking that the CDI system’s inhibition rate dominates toxicity.

## Introduction

Bacteria live almost ubiquitously in complex, spatially structured environments. To survive and prosper against a variety of biotic and abiotic pressures, bacteria must communicate, cooperate, and compete with their surrounding community [1, 2]. Over the last few decades, our understanding of the breadth of strategies used by bacteria for these purposes has increased enormously. They include, for example, communication systems such as quorum sensing [3, 4] and cooperative traits such as diffusible public goods [5]. Alternatively, bacteria can interact antagonistically through the production of toxins to affect competitors [6, 7, 8]. These toxins can be diffusible [9] or require direct contact between the toxin-producing cell and its target cell to deliver toxic effector proteins [10, 11]. Type VI secretion systems (T6SS), for example, mediate killing of neighboring cells through the delivery of bactericidal toxins to a very broad range of target cells upon direct contact [12, 13, 14, 15, 16]. T6SS-mediated killing has been shown to play an important role in the structure and function of bacterial populations [17, 18, 19]. Likewise, contact-dependent inhibition (CDI) systems are also capable of delivery of growth-inhibiting toxins upon contact with target cells [20, 21]. These two-partner secretion (TPS) systems, members of the Type Vb class of secretion systems, are widespread among α-, β- and γ-proteobacteria [21]. Different CDI systems have been shown to be involved in communication, cooperation, or competition within bacterial populations [22, 23, 24, 25, 26]. Although CDI systems are well established as having a role in competition, little is known about how the inhibitory effects upon individual target cells differ between CDI systems or how CDI-mediated growth inhibition affects genetically mixed, spatially structured bacterial populations consisting of target and inhibitor cells.

Within complex, spatially structured populations, prolonged cell-cell contacts are common, and competition between strains is intense [27, 28]. The spatial arrangements of these populations are therefore vitally important to the fitness of their inhabitants; the position of a bacterium within a population dictates availability of essential resources, free space to grow [29], and exposure to external abiotic stresses [30, 31]. Spatial structuring can occur through stochastic process such as genetic drift and bottlenecking, which create strong genetic segregation during microbial range expansion [32]. However, social interactions are able to strongly influence the assortment of bacteria within spatially structured populations. Cooperative traits, such as cross-feeding, promote the intermixing of genotypes [33, 34, 35], with structured environments helping to stabilize populations against the invasion of non-cooperating cheats [36, 37]. In contrast, competition systems such as T6SS [17] or diffusible toxins [38, 39, 40] can reinforce genetic segregation, leading to the formation of clonal patches within a population. Moreover, T6SS may aid bacteria in their ability to invade or defend established natural populations [41, 42]. Likewise, secretion of extracellular matrix can also be used as a weapon to physically displace neighboring cells [43].

CDI systems have been shown to inhibit the growth of target cells within both homogeneous and spatially structured populations [20, 44]. CDI systems typically consist of three genes arranged in a single operon, *cdiBAI*. CdiB encodes a β-barrel outer membrane protein, providing the membrane anchor for the filamentous CdiA that delivers its C-terminal toxin domain into a target bacterium upon contact [45]. CdiI provides immunity, preventing self-intoxication by forming a complex with its cognate toxin and neutralizing its activity [21, 46, 47]. Toxin delivery is receptor dependent: toxins can only be delivered if the target cell expresses the cognate outer and inner membrane receptor proteins for the specific CDI system. These receptors allow the import of the C-terminal CdiA toxin domain (CdiA-CT) into the target cell [45, 48]. Each CDI system is equipped with a single toxin. However, toxin domains are highly divergent between CDI systems, which include tRNase [49, 50], pore-forming [51], or DNase [49] activities. Differences in the CdiA receptor-binding region, which result in different outer membrane receptors being recognized, are used to classify CDI systems [52]. The specificity of Class I and Class II CdiA of *Escherichia coli*, which bind to, respectively, BamA [53] and heterotrimers of OmpC and OmpF [54], limit these classes of CDI to intraspecies toxin delivery. Thus, we define the potency of a given CDI system as the cumulative effect of receptor binding, toxin delivery, and toxin effect, together with level of expression of the system.

Although our molecular knowledge of CDI is improving, our understanding of the impact and effect of the potency of CDI systems on interactions between inhibitor and target strains within spatially structured populations is largely unknown. Mathematical models predict that CDI-like competition systems may result in localized aggregation of inhibiting cells within one- and two-dimensional populations [55]. CDI systems of *Burkholderia* have also been shown to competitively exclude “non-self” from pre-established biofilms and alter the community composition of spatially structured populations [44]. Yet, there has been no link between theoretical predictions of the effect of CDI and experimental data. Furthermore, there is no description and quantification of CDI-mediated cell-cell interactions at the single-cell level. This knowledge will allow for the development of biologically parameterized computational models with the predictive power to identify key parameters of CDI-mediated competition within spatially structured populations. Here, we present the first iterative approach to achieve this goal. Using experimental CDI systems to investigate the single-cell responses of CDI-induced intoxication allowed us to identify key variables of CDI-dependent cell-cell interactions. This in turn facilitated the parameterization of computational models that explore the effect of CDI at a population level, which in turn was validated using the experimental system. Through this iterative approach we identify system-specific factors, including levels of toxicity, timescales of inhibition, and biological cost of CDI systems, that together modulate the outcome of interactions between CDI-expressing cells and susceptible target cells within spatially structured populations.

## Results

### CDI Systems Cause Subtle Growth Retardation on the Single-Cell Level

The growth-inhibiting effect of CDI has been studied extensively at the population level in well-mixed liquid cultures [20, 47, 52]. This approach does not provide detailed information about real-time effects upon cell-cell contact that are crucial to understanding the effect of CDI. Therefore, to assess and quantify the effect of CDI upon contact, we performed competitions between inhibitor and target strains on agarose pads and followed growth of single cells. We engineered two *Escherichia coli* MG1655 inhibitor strains that express CDI from a single-copy, plasmid-based CDI expression system, expressing either the *cdiBAI* operon of *E. coli* EC93 (Class I-Pore-Forming toxin [PFT] CDI system) or *E. coli* UPEC536 (Class II-tRNase CDI system). Each inhibitor strain was competed with an isogenic *E. coli* MG1655 strain lacking the CDI system. The strains were non-motile when growth on the agarose pads. This approach removes differences in the regulation of expression between the systems and isolates any observed effects to all other aspects of CDI potency: the cumulative effect of receptor binding, toxin delivery, and toxin effect.

**Figure 1:**
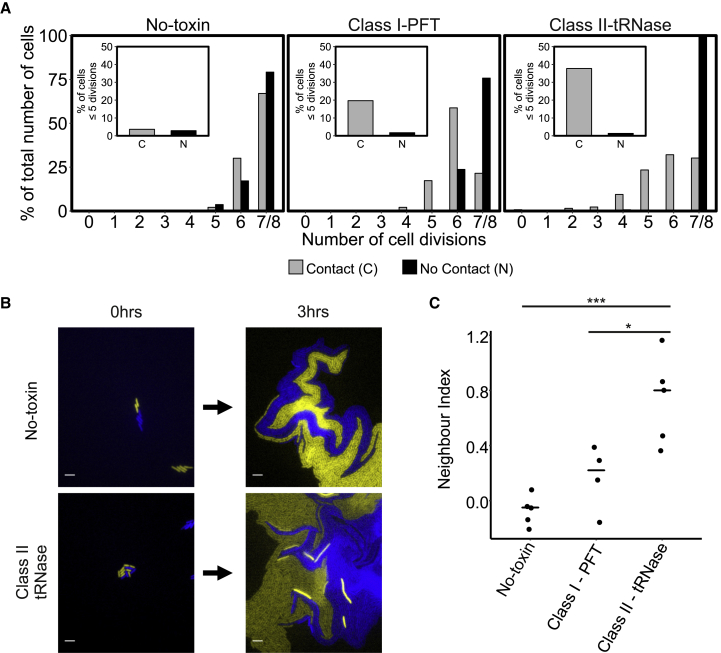
CDI Systems Cause Subtle and Variable Growth Reduction on the Single-Cell Level and Increase the Number of Isolated Target Cells at the Contact Interface (A) Analysis of single cell growth by microscopy (quantified as number of cell divisions) of target cells either in or not in contact with inhibitor cells during a fixed time period of 2 h 33 min. Insets show the total number of target cells with ≤5 cell divisions when either in or not in contact with inhibitor cells. Data were collected from at least 4 interacting microcolonies per system and originated from at least two independent experiments. No-toxin (no contact: n = 382 cells; contact: n = 466 cells), Class I-PFT (no contact: n = 194 cells, contact: n = 335 cells), and Class II-tRNase (no contact: n = 289 cells, contact: n = 265 cells). (B) Representative microscopy images showing begin and end points of interacting fluorescently labeled inhibitor and target cells. The target strain expresses mCherry and is false colored yellow, while the No-toxin and Class II-tRNase strains express msfGFP and are false colored blue. Within 3 h of competition, target cells in contact with inhibitor cells (Class II-tRNase) become isolated from their siblings. Scale bars correspond to 5 μm. See Figures S1 and S2 for membrane integrity staining. (C) The neighbor indices of mixed microcolonies containing inhibitor and target cells following 3 h of growth. CDI system had a significant effect on the neighbor indices of microcolonies (One-way ANOVA, F_3,15_ = 10.68, p < 0.001) with the neighbor index of microcolonies containing Class II-tRNase-encoding bacteria being significantly higher when compared to No-toxin control and Class I-PFT microcolonies (Tukey’s HSD test). Significance levels: ^∗^p < 0.05, ^∗∗∗^p < 0.001. Lines represent the median of 5 No-toxin, 4 Class I-PFT, and 5 Class I-PFT microcolonies. See also Figures S1 and S2 and Tables S1 and S2.

Competition experiments were carried out by inoculating agarose pads with both inhibitor and target cells. Target cell lineages were then tracked using epifluorescence microscopy, and the number of cell divisions over a given period of time, either in contact or not in contact with an inhibitor cell, was measured. When a target strain was in competition with strains that did not contain the *cdiBAI* genes (No-toxin control), approximately 75% of the target cells underwent 7 or 8 cell divisions, and 25% underwent 6 cell divisions, independent of whether contact was made with a No-toxin inhibitor cell (Figure 1A). In contrast, when target cells were in contact with inhibitor cells expressing either of the *cdiBAI* systems, the target cells underwent far fewer cell divisions (Figure 1A). Specifically, the percentage of target cells with an inhibited number of cell divisions (defined as ≤5 cell divisions) increased for cells in contact with inhibitor cells compared to cells not in contact with inhibitor cells (Figure 1A, inset). This difference was more pronounced when in contact with the Class II-tRNase system than with the Class I-PFT system, but both systems exhibited heterogeneity in their effect upon target cell growth. Cell lysis of target cells was very rarely observed when in contact with either of the CDI systems, and membrane integrity was also maintained in the vast majority of target cells (Figures S1 and S2). Together, these results show that the CDI systems tested cause a subtle growth retardation of target cells along the contact interface rather than immediate growth arrest or cell lysis and that the extent of growth retardation is dependent on the type of CDI system expressed. This results in a single-cell-wide boundary of growth-inhibited target cells at the interface between target and inhibitor populations.

To quantify if CDI affected the interface between target and inhibitor cell types, we calculated a neighbor index (see STAR Methods for details), which describes how the proportion of bordering inhibitor and target cells changes between the start and end of the experiment (Figures 1B and 1C). Neighbor indices above 0 indicate that more inhibitors than targets are found at the contact interface at the endpoint of the competition compared to the start. The average neighbor index is significantly higher for competitions with the Class II-tRNase system compared to either the No-toxin control and Class I-PFT system, while the Class I-PFT system has a slightly, but not significantly, higher average neighbor index compared to the No-toxin control (Figure 1C). The increase in neighbor index shows that the organization of cells with the community, and specifically the number of contacts between target and inhibitory cells, can be altered by the presence of CDI within simple two-strain bacterial communities.

### Computational Modeling Shows That Inhibition Rate Has a Larger Effect Than Toxicity on the Outcome of Competition

**Figure 2:**
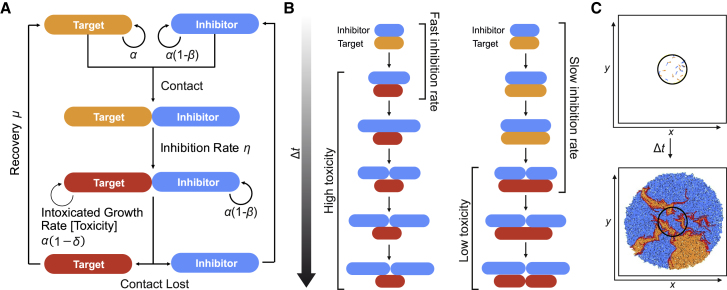
Individual-Based Modeling of CDI within 2D Colonies A dynamic individual-based model is used to simulate how the interactions between CDI-expressing inhibitor cells and their targets alter the growth of bacterial communities. (A) Three types of cells are represented within the model: uninhibited targets (orange), inhibitors (blue) and inhibited targets (red). Target and inhibitor growth rates can be parameterized independently. Contact between the two cell types leads to the inhibition of target growth rate; if contact is lost, the inhibited cell can recover back to its original growth rate. α, cell growth rate; β, cost to inhibitor cell; η, inhibition rate; δ, toxicity; μ, recovery rate. (B) Visual representation of inhibition rate and intoxicated growth reduction; rates are not to scale. Upon contact between a target and inhibitor, a Gibson-Bruck algorithm is used to stochastically determine the time until the target cell becomes inhibited based on the inhibition rate (η). When the target cell becomes intoxicated the growth rate is reduced. (C) Target and inhibitor cells are randomly inoculated within a central circle upon a solid surface at set inoculum densities and equal ratios. Growth proceeds within the model through the elongation and division of the cells, which are represented as rigid rods. Growth is restricted by viscous drag and forces exerted by neighboring cells leading to reduced growth within the center of the colony and competition for space at the expanding edge of the colony. Progressing the simulation forward in time leads to the radial expansion of the colony, with sectoring of the two genotypes occurring as an emergent property of the simulation. See also Table S3.

The single-cell analysis described above was used to develop and parameterize an individual-based model (IBM) to study the effect of CDI expression within larger bacterial populations. Critical values of the model such as cell length, radius, and doubling time, as well as key parameters describing the effect and rate of CDI-induced inhibition, were derived from the single-cell microscopy. The modeling system (outlined in Figure 2A, further details in STAR Methods) was used to explore how the subtle and differential effects of CDI at a single-cell level alter the outcome of CDI-mediated competition within spatially structured bacterial microcolonies. The model describes CDI systems using two parameters: (1) the extent of growth retardation inflicted upon targets, referred within the model as toxicity, and (2) the continuous contact time required before inhibition of targets occurs, referred to as the inhibition rate (Figure 2B). We chose to explore the upper and lower feasible bounds of these parameters within the model, modeling high toxicity (reducing growth rate of intoxicated target cells by 100%) and low toxicity (reducing growth rate of intoxicated target cells by 20%) along with fast inhibition (inhibition rate 1 h^−1^, equating to a mean contact time of 30 min before inhibition) and slow inhibition (inhibition rate 0.1 h^−1^, equating to a mean contact time of 300 min before inhibition). As the single-cell analysis showed heterogeneity in the time taken for inhibition to occur (Figure 1A), we implemented stochastically delayed inhibition of target cells upon contact with inhibitors using a Gibson-Bruck algorithm [56] within the modeling framework CellModeller [57]. Within these simulations, both strains were able to achieve the same maximum growth rate; i.e., expressing the CDI system imposed no cost to the bacterium. Target and inhibitor cells were inoculated at equal ratios but at varying inoculum cell densities within a circle at the center of the simulation (Figure 2C).

**Figure 3:**
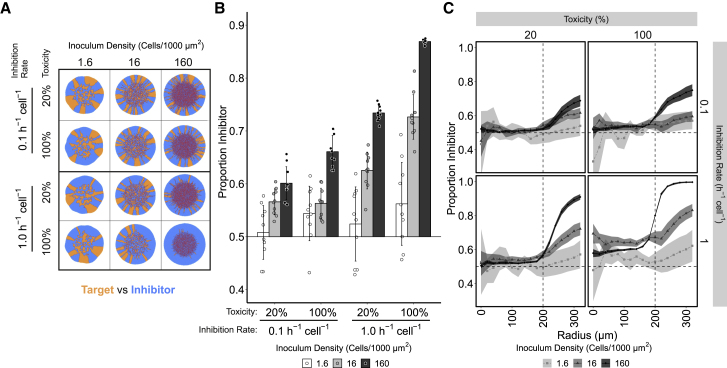
Computational Modeling of CDI Indicates Inhibition Rate Rather Than Growth Reduction of Target Cells Is the Dominant Parameter Determining Outcome of Competition **(**A) Representative output of individual-based modeling capturing the interaction between target (orange) and inhibitor (blue) cells growing in 2D on a solid surface. Inhibited target cells are colored red. The parameters of the CDI system and their effects on population structure were explored at different inoculum densities. The final population size of the simulated colonies was approximately 150,000 cells. (B) The mean ratio of inhibitor (I) to target (T) cells following simulated competition with target cells across inhibition rate and toxicity parameter values and increasing inoculum cell density. The initial ratio of inhibitors within the simulation was 0.5; growth rate of inhibited targets is expressed as percentage of normal growth rate. Both inhibition rate and the intoxicated growth rate had significant and interacting effects on the end-point proportion of inhibitors within the population (robust two-way ANOVA for trimmed means, inhibition rate: Q = 402.08, p < 0.01; toxicity: Q = 128, p < 0.01; interaction: Q = 18.89, p < 0.01), with inhibition rate having the largest effect size (inhibition rate, *ω*^2^ = 0.47; toxicity, *ω*^2^ = 0.12; interaction, *ω*^2^ = 0.16). Error bars represent standard deviation (10 simulations per parameter set) (C) The mean ratio of inhibitor to target cells at increasing radial annuli after simulated competition with target cells across parameter values and increasing inoculum density. Panel labels show intoxicated growth rate and inhibition rate of each simulation. The horizontal dashed line represents the initial ratio of inhibitors within the simulation, and the vertical dashed line represents the radius of the inoculum area. Lines colored by inoculation cell density and shaded areas represent 95% confidence intervals (n = 10). See Figure S3 for no-CDI control simulations. See also Figure S3 and Table S3.

Population growth in the model gave rise to two areas of distinct patterning (Figure 3A and Figure S3A), consistent with mixed strain colonies [32]. In the center of the colony, growth fills local patches within the inoculum area, producing an initially well-mixed population. Once the inoculum area is occupied, the population expands radially, forming the second area of distinct patterning. In this region of the colony, the mixed population transitions into single strain sectors, a well-described phenomenon in microbial colonies [58], and occurred with (Figure 3A) and without (Figure S3A) the presence of CDI in the simulation.

Introducing the CDI parameters into one of the competing strains in the model led to variations in the success of the inhibitor strain as determined by different analyses. First, the presence of CDI increased the proportion of inhibitor cells within the population (Figure 3B), particularly during radial expansion (Figure 3C). The effect of CDI was greatest at high inoculum densities, allowing the inhibitor strain to dominate the population farther from the center of the colonies. However, CDI had little to no effect on the outcome of competition at low inoculum densities. This is in common with other social interactions that are also strongly dependent on inoculum density [31, 59, 60, 61]. Second, to test if a CDI system’s requirement of cell-cell contact was responsible for the observed effect of density dependence, simulations were conducted between two strains that differ in their growth rates, but in the absence of CDI (Figure S3). In this instance, inoculum density did not have a significant effect on the overall ratio of the two strain types at the end of the simulation (Figure S3B), as expected for mixed colonies in the absence of social interactions.

At high inoculum densities, where CDI had the largest effect, the CDI-specific parameters strongly influenced the outcome of competition. The toxicity and inhibition rate had a significant and interacting effect on the proportion of inhibitors in the population at the end of the simulation (“end-point proportion”) (Figure 3B). Specifically, high inhibition rates combined with high toxicity resulted in a larger proportion of inhibitor cells within the population. Of these two system-specific parameters, inhibition rate had the greater effect, meaning that the time taken for inhibition to occur after contact between targets and inhibitors was the dominant parameter of the CDI system determining the outcome of the competition (compare 100% toxicity/0.1 h^−1^ cell^−1^ inhibition rate with 20% toxicity/1.0 h^−1^ cell^−1^ inhibition rate at 160 cells/1,000 μm^2^; Figures 3B and 3C).

**Figure 4:**
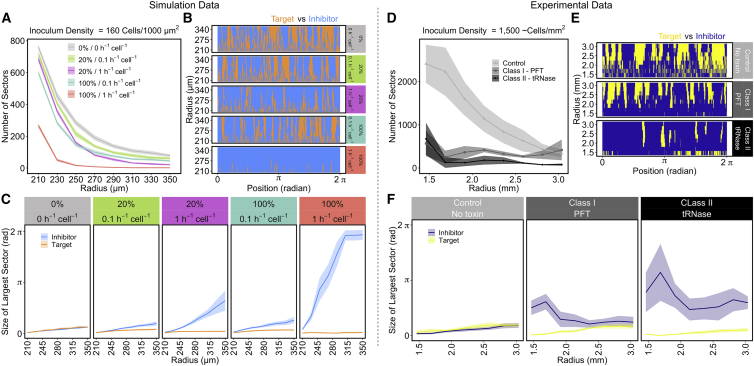
CDI Effect on Sector Frequency and Sector Size during Radial Expansion (A–C) The effect of CDI upon sectoring with high inoculum density (160 cells/1,000 μm^2^) in the computational simulations presented in Figure 3. (D–E) The same sector analyses for experimental data presented in Figure 5 (inoculum density 1,500 cells/mm^2^). (A) The number of sectors formed during radial expansion for each parameter set. Shaded areas represent 95% confidence intervals (n = 10). (B) A representation of the normalized radial expansion zone of simulated colonies presented in Figure 3A. Blue represents inhibitor sectors; orange represents target sectors. Cell positions within each annulus are normalized to the length of the largest annulus and their position plotted as radians around the colony. (C) The size of the largest sectors, plotted as radians, during radial expansion. Shaded areas represent 95% confidence intervals (n = 10). (D) Number of sectors formed during radial expansion in No-toxin control, Class I-PFT, and Class II-tRNase colony competitions. Shaded areas show 95% confidence intervals (n = 6). (E) Representation of normalized expansion zone of colonies presented in Figure 5A. Blue area represents inhibitor sectors; yellow area represents target sectors. (F) The size of sectors formed during radial expansion; shaded areas represent 95% confidence intervals (n = 6). See also Figure S3 and Tables S1–S3.

The two CDI parameters also had different effects on the frequency, size, and survival of target sectors during radial expansion of the colony (Figures 4A, 4B, and 4C). These parameters significantly interact, causing the complete dominance of the inhibitory strain when both inhibition rate and toxicity are high. However, the simulations show that a high inhibition rate combined with low toxicity initially allowed a large number of sectors to emerge from the inoculum area, and the number of sectors decreased rapidly during radial expansion. This is in contrast to low inhibition rate and high toxicity, where fewer initial sectors form and are lost more slowly during radial expansion (Figure 4A, compare 20% / 1 h^−1^ cell^−1^ [purple] with 100% / 0.1 h^−1^ cell^−1^ [cyan]). Ultimately, this results in fewer surviving target sectors at the edge of the colony in high inhibition rate simulations (Figure 4B). High inhibition rates coupled with low toxicity also impacted the size of sectors; larger sector sizes formed during radial growth when compared to high toxicity in combination with low inhibition rate (Figures 4B and 4C). A growth rate difference alone, without the presence of a potent CDI system, did not reduce the number of sectors that emerged from the inoculum area (Figures S3D and S3E).

Simulations show that high inhibition rates have more influence than high toxicity in causing the contraction and eventual extinction of target cell sectors and the propagation of larger sectors of inhibitor cells. Low levels of CDI potency, characterized by low inhibition rate, may thus allow for the maintenance of a mixed population of inhibitors and targets. This is in contrast to simulations in the absence of CDI, which show that coexistence is lost at a 20% difference in growth rate but maintained at lower differences (Figures S3D and S3F). Therefore, the model predicts that for a CDI system to convey a competitive advantage within a spatially structured population, it does not necessarily have to completely inhibit the growth of target cells. Rather, a small reduction in the target cell’s growth rate, effected shortly after contact with an inhibitor cell, may be sufficient for inhibitor strains to outcompete target strains.

### Colony Competitions Are Consistent with Modeling Predictions and Reveal Distinct Potencies of Different CDI Systems

**Figure 5:**
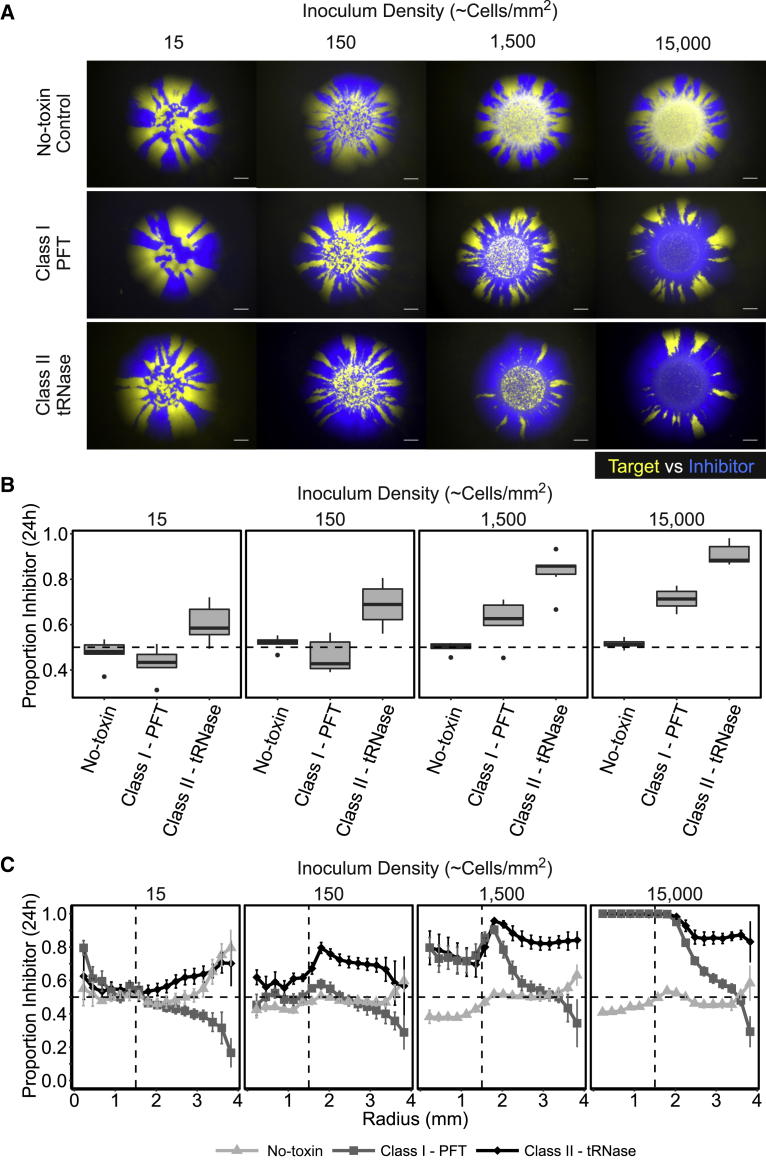
CDI Systems Drive Spatial Segregation at High Inoculum Densities and the Effect Can Vary between Different Systems (A) Representative fluorescent stereomicroscope images of colony competitions between *E. coli* target and inhibitor cells with increasing inoculum density. The target strain expresses mCherry and is false colored yellow, while the No-toxin, Class I-PFT, and Class II-tRNase strains express GFP and are false colored blue. No-toxin results in equal ratios of blue and yellow strains independent of density, whereas CDI systems allow their strain to dominate when at high inoculum densities. Scale bars correspond to 1 mm. Images are representative of 6 replicate competitions, 3 with the CDI system in the GFP background and 3 with the CDI system in the mCherry background. See Figure S4D for representative microcolony images of the latter. (B) The proportion of inhibitor cells (or blue cells in the No-toxin control) against target cells as determined by fluorescence (see STAR Methods) within colonies after 24 h of competition with increasing inoculum density. Boxplots show median, 25^th^/75^th^ percentiles, 1.5 ^∗^ IQR, and outliers (n = 6). There was a significant and interacting effect of both CDI system and density on the end-point proportion of inhibitors in the populations (ANOVA, F(6,84) = 9.197, p < 0.01). Across all tested densities, Class II-tRNase system increased the end-point proportion of inhibitors to a greater extent than Class I-PFT system (Post-hoc Tukey’s Tests, all p < 0.05) (C) The proportion of either No-toxin control (light gray) Class I-PFT (dark gray), or Class II-tRNase (black) cells against target cells at increasing radial annuli after 24 h of competition within colonies. Dashed vertical line represents approximate inoculum radius. Error bars represent SEM (n = 6). (B and C) The dotted horizontal line represents the initial ratio of inhibitor cells (blue cells in the No-toxin control) within the inoculum. See also Figures S4 and S5 and Tables S1 and S2.

As our IBM predicts that different CDI systems may have different effects on the outcome of competition within spatially structured populations, we sought to test key model predictions empirically. Mixed communities consisting of equal ratios of CDI-expressing inhibitor *E. coli* and sensitive target *E. coli* were inoculated on agar plates (Figure S4). Inhibitor cells expressed a complete CDI system, whereas target cells expressed an incomplete system lacking the C-terminal toxin of CdiA and immunity protein (CdiI) to confer susceptibility to CDI toxins delivered upon contact with an inhibitor cell. Consistent with the modeling, initial growth of the cells fills the central inoculum area before population growth transitions to radial expansion independent of the presence of CDI. During expansion, the two strains segregate into sectors at the outer edge of the colony (Figure 5A).

We see an effect of CDI predominately in the radially expanding area. However, as also predicted by the modeling, the experimental CDI systems showed that the end-point proportion of inhibitor cells is strongly dependent on the inoculum density. *E. coli* MG1655 cells expressing either the Class I-PFT or Class II-tRNase CDI systems were only able to outcompete target cells when the inoculum density was high (Figures 5A and 5B). In contrast, low inoculum cell densities did not result in a significant deviation from the initial equal ratio of inhibitor to target. Therefore, both the modeling and experimental data show that the effects of CDI within spatially structured populations are greatest at high inoculum densities.

Although both CDI systems have a significant effect on the outcome of the colony competition, the competitions also reveal that the two CDI systems examined have different potencies. Expression of the Class II-tRNase CDI system resulted in a significantly higher end-point fraction of inhibitory cells than the Class I-PFT CDI system across all densities (Figure 5B). Moreover, although both CDI systems greatly reduced the number of sectors that emerge from the inoculum area when compared to the No-toxin control treatment (Figure 4D), the sector sizes of Class I-PFT inhibitor cells shrink toward the edge of the colony (Figures 4E and 4F). This resulted in a reduction in the fraction of Class I-PFT-expressing inhibitor cells as the colony radially expanded (Figure 5C). In contrast, the Class II-tRNase CDI system supported larger inhibitor sector sizes during range expansion (Figures 4E and 4F), which resulted in a greater proportion of inhibitory cells at the outer edge of the colony (Figure 5C). Counterintuitively, the sectors formed by target cells in competition with Class-I PFT CDI-expressing inhibitors broaden during expansion. This occurs despite the initial competitive success of the CDI-expressing strain (Figures 5A and 5C), which is indicative of differential growth rates between the competing strains [32]. Together, the results of the colony competition assays show that CDI systems can affect the segmentation of radially expanding bacterial colonies. Furthermore, our model prediction, that differences between CDI system parameters can lead to different spatial outcomes, is valid. In summary, the outcome of CDI-mediated competition within colonies is strongly dependent upon when contact between inhibitors and targets is established--governed by the initial density of cells–and the potency of the CDI system being expressed.

To further test how CDI potency affects the outcome of competition, and to examine the effect of mutual inhibition within colonies, competitions were conducted between Class I-PFT and Class II-tRNase expressing *E. coli*. Across all densities tested, expression of Class II-tRNase provided cells with a significant competitive advantage over Class I-PFT expressing cells (Figures S5A and S5B). As with the previous competitions, the increase in proportion was particularly pronounced during radial expansion (Figure S5C). The resulting proportion of Class II-tRNase cells in the populations is comparable to that observed when competed with a target lacking the C-terminal toxin of CdiA and immunity protein (Figure 5). This suggests that bacteria expressing the Class I-PFT CDI system are not able to mitigate the competitive advantage of cells expressing the more potent Class II-tRNase CDI system.

### Costs Induced by CDI Influence the Outcome of Competition

**Figure 6:**
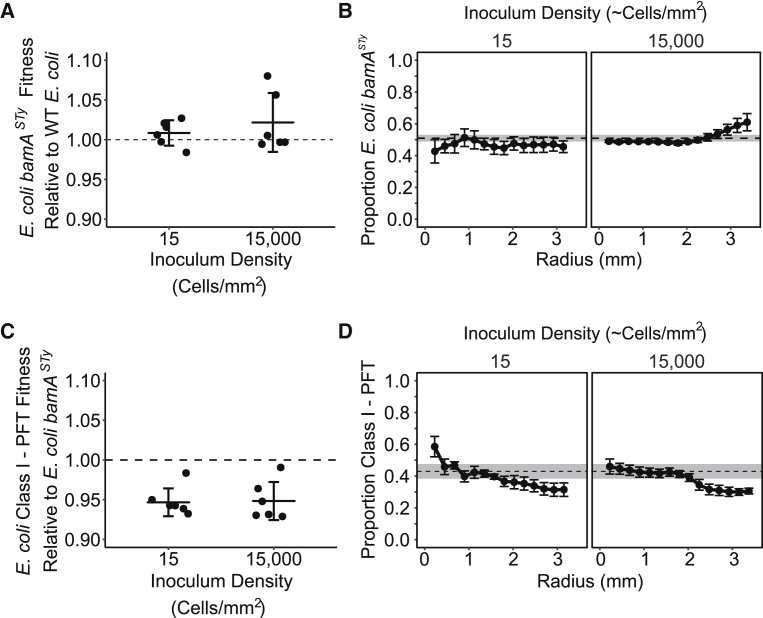
Expression of the Class I-PFT CDI System Carries a Cost to *E. coli* When Competing within Spatially Structured Populations (A and B) Replacement of WT *E. coli bamA* with *S. enterica bamA* does not significantly alter the relative fitness of *E. coli* within colonies. Colony competitions between WT *E. coli* and *E. coli* expressing *bamA*^*STy*^. (A) The fitness of *E. coli bamA*^*STy*^ relative to WT *E. coli*. The relative fitness is not significantly different from 1 at high (one-way t test, t_5_ = 1.4309, p = 0.21) or low (one-way t test, t_5_ = 1.3007, p = 0.25) inoculum density. (B) The radial distribution of *E. coli bamA*^*STy*^ relative to *E. coli* throughout the mixed colony. Points represent mean of 5 replicates, two with the *E. coli bamA*^*STy*^ in a marker GFP background and 3 with *E. coli bamA*^*STy*^ in a mCherry marker background. (C and D) Colony competitions between *E. coli* harboring Class I-PFT CDI system and *E. coli bamA*^*STy*^. (C) The fitness of *E. coli* Class I-PFT relative to *E. coli bamA*^*STy*^. The Class I-PFT CDI system imposed a significant ∼5% cost on *E. coli* when inoculated at both low density (one-sample t test, μ = 1, t_5_ = −5.2702, p < 0.01) and high density (one-sample t test, μ = 1, t_5_ = −7.46, p < 0.001). (D) The radial distribution of *E. coli* Class I-PFT relative to *E. coli bamA*^*STy*^ throughout the mixed colony. (A and C) Points represent independent replicates, horizontal line shows the mean, and error bars show the standard deviation (n = 6). The horizontal dashed line represents equal fitness. (B and D) Points represent mean of 5 or 6 replicates, and error bars represent SEM. Horizontal dashed line indicates initial inoculum ratio and gray shaded area represents SEM (n = 6). See also Tables S1 and S2.

As the colony competitions between Class I-PFT-expressing inhibitor cells and their targets suggested that there was a disparity in the growth rates between the two strains, we sought to quantify the cost of expressing this CDI system. To achieve this, we competed *E. coli* cells harboring the Class I-PFT CDI system against a resistant receptor mutant, *E. coli* expressing a non-cognate *Salmonella enterica* BamA (BamA^STy^). This isolates any potential growth disadvantage imposed by the CDI system upon inhibitors from the effect of inhibition of the target, as delivery of the CDI toxin by the *E. coli* Class I system does not occur to cells expressing non-cognate BamA [22, 62]. We first tested the relative fitness of the *E. coli bamA*^*STY*^ strain compared to the parental *E. coli* strain and observed no difference in its relative fitness (Figures 6A and 6B). In contrast, the Class I-PFT CDI system imposed a significant 4.9% cost upon the expressing strain independent of inoculation density (Figure 6C). In addition, the fraction of Class I-PFT CDI cells decreased at the edge of the colony (Figure 6D), suggesting that the effect of the cost of expressing the CDI system is predominantly during the radial expansion phase. A different approach was used to assess the cost of the Class II-tRNase CDI system in the absence of an amenable receptor mutant. Instead, the cost was approximated through competition against *E. coli*-expressing CdiI immunity protein of UPEC536 and suggested that the Class II-tRNase CDI system imposed a lower net cost of 3.3% upon the expressing strain (data not shown).

**Figure 7:**
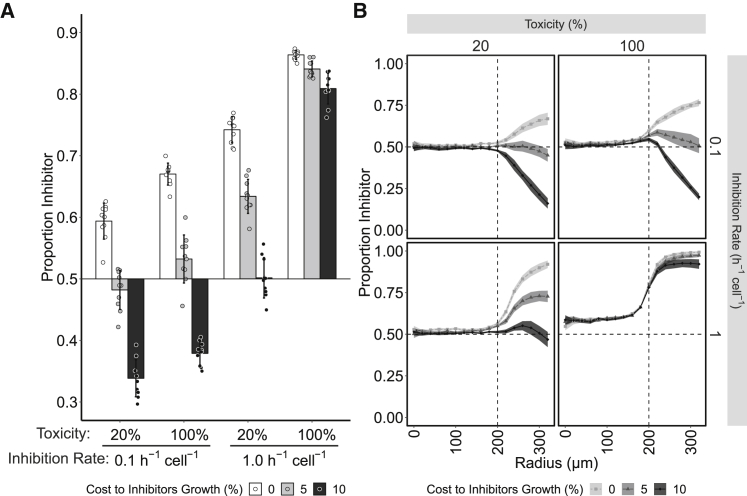
Computational Modeling Predicts That Costs Imposed upon Inhibitors by CDI Systems Can Outweigh the Benefit when the Potency of CDI Is Low A cost to the inhibitor cells was imposed as a 0%, 5%, or 10% reduction in growth rate, and simulations were then run with the same parameters as in simulations presented in Figure 3. Shown are results of simulations at high cell-density inoculum (160 cells/1,000 μm^2^). For low inoculum density simulation results, see Figure S6. (A) The proportion of inhibitor cells following simulated competition with target cells at different inhibition rate and toxicity parameter values. Bars are colored by the level of cost imposed by the CDI system upon the inhibiting cells. Error bars represent the standard deviation (10 simulations per parameter set). (B) The proportion of inhibitor cells at increasing radial annuli after simulated competition with target cells. Panel labels show toxicity and inhibition rate of each simulation. Horizontal dashed line represents initial inoculum ratio of inhibitors; vertical dashed line represents initial inoculum radius. Lines colored by the cost of CDI and shaded areas represent 95% confidence intervals (n = 10). See also Figure S6 and Tables S2 and S3.

Model predictions were substantively validated by the experiments, but we noted that the model did not predict that target cells could outcompete inhibitor cells during radial expansion, as observed with the Class I-PFT CDI system (Figures 5A and 5C). To test if this could be due to the cost imposed by CDI expression, a growth rate disadvantage to inhibitor cells was added to the model (Figure 7). Simulations showed that a 5% reduction in growth rate of inhibitor cells relative to the target cells could negate the benefit provided by CDI systems when the rate of inhibition was slow, independent of high toxicities (5% and 10% cost, inhibition rate 0.1 h^−1^ cell^−1^ Figure 7A). However, the costs imposed in the model were overcome when inhibition rates were rapid, even when the level of toxicity was low (5% and 10% cost, inhibition rate 1.0 h^−1^ cell^−1^ Figure 7A). The model predicts that the cost of CDI is most evident during radial expansion causing the ratio of inhibitor cells to decrease at the edge of the population. These results closely resemble the experimental quantification of the Class I-PFT cells when growing within a mixed colony where the inhibitor-to-target ratio likewise dips at the outer edge of the colony (Figure 5C). The simulations demonstrate that through implementing a cost in growth rate to inhibitor cells, our model more accurately reproduces the experimentally observed population dynamics of the Class I-PFT CDI system. These results are consistent with the experimental results, which show that costs associated with the expression of CDI systems can strongly influence the outcome of CDI-mediated competition within spatially structured populations. Taken together, the results show that both inoculum density and CDI-specific parameters altering the potency and cost of expression influence and modulate the effectiveness of CDI systems.

## Discussion

Bacteria commonly live within dense, complex, microbial communities. Antagonistic interactions between individuals within these communities are a major contributor toward the community’s composition, structure, and organization [63]. These ultimately affect both the fitness of individuals within the community and the community’s functioning as a whole. CDI systems are a distinct subset of Type V secretion systems that deliver growth-inhibiting toxins upon contact with closely related bacteria. These systems are widespread in gram-negative bacteria and are particularly common in pathogens [47, 64], showing heterogeneity in their structure and toxicity. CDI systems have been shown to alter aggregation, stress resistance through persister cell formation, and competitive exclusion of target cells from bacterial populations [25, 65, 66]. We aimed to determine the extent to which different CDI systems of *E. coli* were capable of influencing the growth of mixed spatially structured populations consisting of targets and inhibitor cells and specifically identify key factors that determine the outcome of CDI-mediated competition within these simple, synthetic two-strain communities.

Using an iterative approach, combining computational methods and synthetic biology to explore CDI systems, we identified that different CDI systems have different potencies that alter the extent to which neighboring target cells are inhibited. Although the effects of CDI on target cell growth rate are subtle, they are sufficient to drive significant, and CDI system-specific, differences in the composition and spatial organization of expanding bacterial populations. The final proportions of cells in CDI-mediated competition was dependent upon the initial population structure (i.e., inoculation cell density)–similar to other social interactions within bacterial communities [31, 59, 60]–but not where there is a fixed growth difference. The results in Figure 4 also indicate a shift in patch size distribution induced by CDI-mediated interactions. The effects of growth differences are insensitive to initial density because they are interaction independent, whereas CDI-mediated social interactions are dependent on cell-cell contact, which are more frequent at high cell density. The outcome of competition is also affected by the biological properties of the CDI system, which we encapsulate by the system-specific parameters of inhibition rate, toxic effect of CDI systems, and cost of CDI. The results of our iterative methodology show consistently that these variables together modulate the extent to which CDI can influence a growing bacterial population.

Many factors act in combination to determine the benefit of CDI systems during bacterial competition. This includes, but is not limited to, the genetic background of wild-type strains, which may encode multiple competition systems (e.g., T6SS, different CDI systems) and the growth condition of the bacteria, which will influence the expression of CDI. Here, we took a reductionist approach to isolate the effect of a single CDI system on the spatial structure of a bacterial population. Thus, experiments were carried out using isogenic strains that only differ in the encoded *cdi* genes that are expressed from the same promoter. We compared two distinct CDI systems and assess the differential impact captured by the CDI potency factors that vary in this experimental setup: toxin activity, toxin import pathway, receptor binding affinity, receptor and CDI surface distribution, CDI turnover, and biological cost of CDI expression, owing to either the metabolic loads associated with CDI [55] or due to self-intoxication [65]. Our data indicate that these variables can be described using three simple parameters, the inhibition rate (contact time required for intoxication of target), the toxic effect, and cost of expression of the CDI system. Our *in silico* and experimental analyses indicate that in our model system, the inhibition rate, rather than the extent of toxicity, has a larger effect upon the outcome of competition between target and inhibitor strains. This is because fast inhibition rates during radial growth prevent the expansion of target sectors. In the absence of fast inhibition, the effect of a highly toxic system is diminished due to the protection provided by the sectoring of the colony. The rate of inhibition is a biologically relevant parameter and will be determined by variation in CDI assembly, receptor binding affinity, and toxin import pathways, among others. If inhibition is sufficiently rapid, even a slight reduction in growth of the target cells can still benefit the inhibitor strain. These results show that CDI systems do not necessarily need to kill their target cells to gain a benefit within a spatially structured environment.

The results show that the effect of CDI is dependent on the cell density of the inoculum. This dependence on inoculum density is a feature found in other studies of a wide variety of different competitive interactions [31, 59] and is attributed to a close relationship between competition and spatial patterning. Population density is also a critical factor in direct competition systems; for example, many diffusible toxins are produced in a density-dependent manner, allowing for a coordinated attack [67]. The CDI systems studied here provide an alternative mechanism of dependence on density: low inoculum cell densities allowed target subpopulations to form patches that provided protection to a substantial proportion of the susceptible target population through physical isolation of the two cell types. In contrast, high inoculum cell densities provided a greater opportunity for more contacts to be formed and for early inhibition of target cells, leading to inhibition of the target population before protective patches were formed. The important role of the cell-density-dependent contact for CDI is further illustrated by the absence of a cell density effect on the proportion of target cells when a 20% growth rate difference was introduced between two strains in the absence of CDI.

As CDI is also dependent on receptor specificity, and consequently appears to be predominantly an intraspecies competition system, any emergent patterning will create defined boundaries within any mixed-strain but single-species population. We show that CDI generates non-growing, single-cell-wide boundaries of the target strain that will provide protection to further inhibition of target cells. Therefore, strains harboring these Type Vb CDI systems are not likely to displace pre-existing target populations, unlike some T6SS, which have been shown to be able to displace resident communities [15, 19]. The benefit of CDI as illustrated here thus reflects a specific natural situation where neither population was fully established at the start, and that was modeled in the lab with our experimental system and simulations.

We have shown that CDI systems are capable of altering the composition of expanding bacterial colonies through limiting the number of sectors that are able to emerge from inoculum zone. However, different CDI systems lead to different sectoring patterns due to a combination of potency and cost of expression. These differences between CDI systems may provide differential benefits in different environmental conditions. Intriguingly, many bacterial genomes encode multiple distinct CDI systems along with different competitions systems, such as T6SS and diffusible toxins that are likely to be differentially expressed [50, 68, 69]. Moreover, the toxicity of CDI systems may be modulated through recombination between the toxin tip and immunity and the array of orphan toxins and immunity genes that are often found downstream of CDI systems [70]. Understanding the control of expression within variable environments may shed further light on the impact and roles of different CDI systems and related potency. Moreover, as CDI systems can occur in a wide variety of genomic contexts [66, 70, 71], it is clear that the specific benefit of harboring CDI must be placed within its genomic context to be fully understood.

In contrast to T6SS, where mutual antagonism is required for the formation of stable mixed populations [17, 18], both unilateral and mutual CDI inhibition led to mixed-strain populations. When T6SS-mediated killing is unilateral, T6SS null strains are effectively eliminated from randomly mixed populations of *Vibrio cholerae* or *Aeromonas hydrophilia* [18, 72, 73]. This difference between T6SS and CDI is likely due to the differential toxic effect of the two competition systems. Whereas T6SS deliver toxic effectors that are often able to kill and lyse neighboring target cells, we have shown that the *E. coli* CDI systems studied do not cause immediate cell lysis or death. Rather, CDI systems have a more subtle effect, inhibiting the target cell growth rate and creating single-cell-wide boundaries between populations. It is remarkable that CDI systems with their subtler impacts and inability to “deal death” are able to generate qualitatively similar outcomes to T6SS at a population level [17]. Any emergent population structure has implications for other cooperative traits: for example, CDI has been

implicated in biofilm formation through adhesion between CdiA stalks [22, 74], cell-cell signaling through modification of gene expression [24], and the formation of persister cells [65]. There is likely no single role of CDI; any modifications of spatial structure, even subtle ones, may provide multiple impacts within different environments.

This study adds to a growing body of work demonstrating the role of both intra- and interspecies competition systems in modulating the composition of spatially structured microbial communities [17, 44, 63]. Unlike other competition systems [18, 67], we show here that the subtle growth inhibitory effect of CDI systems generates single-cell-wide boundaries between strains, which are sufficient to impact population composition. This insight may provide a new way to engineer synthetic bacterial communities and contribute to understanding of natural mixed communities. Future work is required to determine how CDI systems, along with other contact-dependent toxin delivery systems and diffusible toxins, modulate spatial structure within more complex environments. This will be particularly important in communities in which multiple systems, including competition systems, are likely to work either synergistically or antagonistically to contribute toward any emergent spatial structure.

## STAR★Methods

### Key Resources Table

**Table:** 

REAGENT or RESOURCE	SOURCE	IDENTIFIER
**Bacterial Strains (lab strain identifier in parentheses)**		

*Escherichia coli* K12 MG1655 (MV784)	Laboratory collection	N/A
*Escherichia coli* K12 MG1655 *attB::Km-gfp* (MV1463)	This paper	N/A
*Escherichia coli* K12 MG1655 *attB::Km-mCherry* (MV1488)	This paper	N/A
*Escherichia coli* K-12 DH5α [genotype F^–^ φ80*lacZ*ΔM15 Δ(*lacZYA-argF*) U169 *endA1 recA1 hsdR17 deoR thi1 supE4412 gyrA96 relA1 λpir*] (MV485)	Laboratory collection	N/A
*Escherichia coli* K12 MG1655 *bamA*^*STy*^*-frt-Km-frt* (MV1793)	This paper	N/A
*Salmonella* Typhimurium LT2 (MV-P86)	Laboratory collection	N/A
*Salmonella* Typhimurium LT2 *bamA-frt-Km-frt* (MV-P1077)	This paper	N/A

**Chemicals, Peptides, and Recombinant Proteins**		

SYTOXblue	Invitrogen	Catalogue number S11348
LB Broth Lennox	Fisher Scientific Ltd.	Catalogue number BP1427-2
Ampicillin	Sigma	Catalogue number A9518-25G
Kanamycin	Sigma	Catalogue number K1876-5G
Chloramphenicol	Sigma	Catalogue number C0378-25 g
L-arabinose	Sigma	Catalogue number A3256-100G
IPTG	Sigma	Catalogue number I6758-5G
Agarose, molecular grade	Eurogentec	Catalogue number EP-0010-05
Fosmid kit /specific chemicals	Lucigen	Catalogue number CCFOS110 or sub-orders thereof

**Deposited Data**		

Raw data used to produce figures	This paper	https://doi.org/10.6084/m9.figshare.9546731.v1

**Recombinant DNA**		

Primers- see Table S1.		N/A
Plasmids- see Table S2		N/A

**Software and Algorithms**		

Python 2.7.13	Python Software Foundation	https://www.python.org
R 3.5.3	The R Foundation	www.r-project.org
ImageJ 1.51	LOCI, University of Wisconsin	http://www.imagej.net/
Custom Cellmodeller CDI implementation	This paper	https://www.github.com/mbottery/CDI_cellmodeller

**Other**		

Gene Frames 65ul	Fisher Scientific	Catalogue number 11570294
Gene Frames 125ul	Fisher Scientific	Catalogue number 11560294

### Lead Contact and Materials Availability

Further information should be directed to the Lead Contact, Marjan van der Woude (marjan.vanderwoude@york.ac.uk). Requests for plasmids or other reagents or resources should be directed to and will be fulfilled by the Lead contact. This study did not generate other new unique reagents.

### Experimentel Model and Subject Details

#### Bacterial strains and growth conditions

Bacterial strains and plasmids used throughout this study are listed in Key Resources Table. Bacteria were cultured in Lysogeny Broth (LB) medium (Fisher) unless differently specified. Cultures were grown at 37°C under well-aerated conditions. As relevant, the following chemicals (Sigma-Aldrich) were added to the growth medium to the indicated final concentrations: ampicillin (100 μg/mL; Ap^100^), chloramphenicol (30 μg/mL; Cm^30^), kanamycin (50 μg/mL; Km^50^), L-arabinose (0.2%) and IPTG (1 mM).

### Method Details

#### Construction of bacterial mutants

All *E. coli* and *Salmonella* Typhimurium mutants were constructed using λ red mediated homologous recombination [75]. In short, the relevant bacterial strains were first transformed with the pKD46 plasmid, which contains the λ red genes under an arabinose inducible promoter. Next, bacterial cultures were grown overnight at 30°C (in order to maintain the pKD46 plasmid) and subsequently diluted 1/100 in fresh medium. After 2 h30 incubation at 30°C, 0.2% arabinose was added to the cultures and further left to incubate for 30-60 min. Bacterial cultures were then put on ice and washed 3 times with ice-cold MQ water using a cooled centrifuge. Finally, 5 μL of purified PCR products (around 500 ng/μL) were added to 100 μL of the cell suspension and subjected to electroporation (GenePulser, BioRad). Cells were left to recover for 3-5 h at 37°C and subsequently plated out on agar plates containing the relevant antibiotic and incubated at 37°C, to cure the strains from the pKD46 plasmid. Correct integration of PCR products was further verified by sequencing (Eurofins, Mix2Seq). All primer sequences for constructing the bacterial mutants are listed in Table S1. The *frt*-flanked antibiotic cassette could be removed as previously described [76]. Briefly, the relevant bacterial strains were transformed with the pCP20 plasmid, which consitutively expresses the flippase enzyme, and grown at 30°C in order to maintain the plasmid. The different loci targeted were then PCR-validated and sequence-verified. Finally, bacterial strains were grown at 37°C to cure them from the pCP20 plasmid.

#### Construction of plasmids

Plasmid pDHL1029-*mScarlet* (pMV371) was constructed as follows; primers are listed in Table S1. First the mScarlet gene was PCR amplified from pmScarlet-C1 using oMV1367 and oMV1368, after which the resulting PCR product was digested with EcoRI and XmaI. Next the pDHL1029-msfGFP backbone was PCR amplified using oMV1373 and oMV1374, after which the resulting PCR product was digested with EcoRI and XmaI. Finally, the fragments were mixed together and ligated to make pDHL1029-*mScarlet* (pMV371). The final plasmid was PCR and sequence verified. All enzymes used were supplied from NEB.

All of the remaining plasmids created in this study (pCC1FOS derivatives) were constructed using λ red mediated homologous recombination [75], using exactly the same protocol as described above, in strain EPI300 (Lucigen). Correct integration of PCR products was further verified by sequencing (Eurofins, Mix2Seq). All primer sequences for constructing the pCC1FOS derivatives are listed in Table S1. The *frt*-flanked antibiotic cassette could be removed as previously described [76] and using the protocol described above. All the different loci targeted were PCR-validated and sequence-verified. Correct constructs were isolated from EPI300 after copy control induction, and transformed to the relevant background for competition assays.

#### Fluorescence microscopy and image analysis

Time-lapse fluorescence microscopy experiments were performed with a temperature controlled LSM510 inverted microscope (ZEISS) equipped with, a GFP filter (BP470/40, FT495, BP525/50), a CFP filter (BP436/20, FT455, BP480/40), an mCherry filter (BP560/40, FT585, BP630/75), an X-Cite 120 mercury lamp (Excelitas technologies), and a Zeiss AxioCam HRm camera.

The target strain used was *E. coli* MG1655 derivative MV1488 and the inhibitor strains used were *E. coli* MG1655 (MV784) bearing either plasmid pMV531 (No-toxin control), pMV476 (Class I-PFT CDI system) or pMV485 (Class II-tRNase CDI system). For imaging, targets and inhibitors were grown to exponential phase in LB medium (supplemented with IPTG and Cm^30^ for the inhibitor strains), mixed at a final dilution of 1/40-1/80 and pipetted (1 μL) on LB agarose pads (2% agarose (Eurogentec) concentration, as described [77]). The competition mixtures were left to air dry, after which the system was sealed using Gene Frames (65ul for time lapse, 125ul for snapshots) (Thermo Fisher Scientific) by placing a coverslip on the pad, effectively sandwiching and immobilizing the cells. The microscope slides were placed under the microscope in a heated chamber at 37°C and left to grow while images were taken automatically every 3-10 min (depending on the experiment) for 3-5 h. Exposure times were between 100-500 ms (depending on the fluorophore) and the power of the mercury lamp was used at 75% of its total power (120W). Data was gathered from two independent experiments and from at least 4 interacting microcolonies, and pooled. For the Sytox Blue experiments, SytoxBlue (Invitrogen) was added to the LB agarose at a final concentration of 0.5 μM and fluorescence was detected using the CFP filter.

Images were acquired using AxioVision software (ZEISS) and the resulting pictures were further handled with the open sourced software ImageJ (https://imagej.nih.gov/ij/). Single cell competitions were analyzed as follows. First, only microcolonies where targets and inhibitors were in contact from the start of the experiment (45 min after the cells were put on the pad) were included in the analysis and targets (expressing Cherry) and inhibitors (expressing msfGFP) were discerned based on their fluorescent signal. Next, all target cells that stayed in contact with inhibitor cells during a fixed time of 2 h 33 min (after this time point cells began to grow in 3D and single cell resolution was quickly lost) were tracked and the number of cell divisions they went through was determined. Target cells that lost contact during this time interval or target cells that came into contact in a later time stage were not included in the analysis. For the no contact conditions target cells were tracked that were not in contact during the same fixed period of time and again the number of cell divisions they went through was determined. Most target cells that were not in contact underwent 7 or 8 divisions and this group was pooled and defined as the 7/8 subpopulation. This was a result of our quantification approach, which imposes an arbitrary endpoint and makes it impossible to accurately discriminate between these two subpopulations.

Neighbor indices were calculated by first counting the number of target and inhibitor cells that were in contact at the start of the experiment $\left(t_{0}\right)$ and then counting the number of target and inhibitor cells that remained in contact at the end of the experiment ($t_{end}$, 2 h 33min):

$$\text{Neighbour index}=\log_{2}\left({\mathrm{Inhibitors}}_{t_{\mathrm{end}}}/{\mathrm{Targets}}_{t_{\mathrm{end}}}\right)-\log_{2}\left({\text{Inhibitors}}_{t_{0}}/{\text{Targets}}_{t_{0}}\right)$$

#### Colony competition assays

All experiments were carried out using *E. coli* MG1655 derivatives MV1463 and MV1488 as the strain backgrounds. For the No-toxin control experiment, strain MV1463 bearing plasmid pMV531, was competed with strain MV1488 bearing plasmid pMV532. Target strains for the Class I-PFT CDI system were strain MV1463 bearing plasmid pMV535, and strain MV1488 bearing plasmid pMV536, which were competed with the inhibitor strains MV1488 bearing plasmid pMV533, and MV1463 bearing plasmid pMV476, respectively. Target strains for the Class II-tRNase CDI system were strain MV1463 bearing plasmid pMV537, and strain MV1488 bearing plasmid pMV538, which were competed with the inhibitor strains MV1488, bearing plasmid pMV534 and MV1463 bearing plasmid pMV485, respectively.

Target and inhibitor strains were grown to exponential phase, mixed at equal ratio and serially diluted (tenfold) up to a dilution of 10^−3^ to generate different inoculum densities. 1 μL of the competition mix was spotted on a LB agar plate supplemented with IPTG and Cm^30^ at these different initial cell densities and incubated at 37°C for 24 h to form a colony biofilm (Figure S4A). Under these growth conditions the *E. coli* strains were non-motile. Absolute cell densities per square mm were calculated by quantifying the initial number of cells spotted (determined by CFU) and using the surface area of 6.3 mm^2^, based on the measured radius of 1.4 mm of the 1 μL spot.

#### Image acquisition and quantification

Images of the spots were captured using a stereomicroscope (ZEISS Stereo Lumar V12) equipped with a GFP filter (Excitation BP470/40, Emission BP527/50), mCherry filter (Excitation BP545/25, Emission BP605/70), an AxioCam HRm camera and AxioVision software (ZEISS). Images were handled with ImageJ and false colored blue (for msfGFP expressing strains) and yellow (for mCherry expressing strains).

The ratio of targets and inhibitors along with their spatial distribution within each spot was quantified through image analysis as follows. A manual threshold was applied to each color channel independently to remove background noise and to identify sectoring. The color channels were then merged and the ratio of green to red pixels within the image was measured through comparison of their color intensities based on a minimum color difference threshold. If this threshold was not met the color of the pixel was deemed indistinguishable between green and red. The ratio of the two pixels colors was used as an estimate of the inhibitor to target ratio. To ensure that quantification was not biased by the nature of the fluorophore used, all competitions were conducted with the inhibitor and target strains in both fluorescent backgrounds. In total six independent replicate competitions were conducted, three with the inhibitor in the GFP background and three with the inhibitor in the mCherry background.

#### Relative fitness measurements

The strains used to assess the relative fitness of strain MV1793 were strain MV784 bearing either plasmid pMV531 or pMV532 competed against strain MV1793 bearing either plasmid pMV532 or pMV531, respectively (see Key Resources Table). The strains used to assess the total cost of the Class I-PFT CDI system were strain MV1793 bearing either plasmid pMV531 or pMV532 competed against strain MV784 bearing either plasmid pMV533 or pMV476, respectively. The strains used to assess the total cost of the Class II-tRNase CDI system were strain MV1463 bearing either both pMV531 and pDAL776 or both pMV485 and pBR322, competed with MV1488 bearing either both pMV532 and pDAL776 or both pMV534 and pBR322.

Relative fitness of the strains during spot competitions was measured by first quantifying the initial absolute cell density in 1 μL by plating out on LB with Cm^30^ (the strains of interest were differentially labeled with msfGFP or mCherry and the colonies were counted using the stereomicroscope based on their fluorescent signal). After 24 h of competition, the agar containing the spot of interest was cut out, put in 5 mL of M9 salts and, after vortexing thoroughly, the mixture was plated out on LB with Cm^30^ to quantify the endpoint $\left(t_{24hr}\right)$ cell densities using the stereomicroscope. In total six independent replicate competitions were conducted, three with strain 1 in the GFP background and strain 2 in the mCherry background, and three with strain 2 in the GFP background and strain 1 in the mCherry background. Relative fitness was then calculated as follows [78]:

$$\text{Relative fitness}=\frac{\ln\left(\mathrm{Strain}1_{t_{24\mathrm{hr}}}/\mathrm{Strain}1_{t_{0\mathrm{hr}}}\right)}{\ln\left(\mathrm{Strain}2_{t_{24\mathrm{hr}}}/\mathrm{Strain}2_{t_{0\mathrm{hr}}}\right)}$$

#### Individual-based modeling of CDI

The individual-based model of CDI within bacterial colonies was based on CellModeller [57], an open source multicellular bacterial biophysics modeling framework, integrated with an exact stochastic simulator based on the Gibson-Brock next reaction method [56].

Bacteria within the model are simulated as rigid capsules within continuous three-dimensional space. Each bacterium is described by its length, radius, position, orientation, cell type and growth rate. Cells grow through exponential elongation at their poles, with the unconstrained growth being proportional to their length: ΔL=α⋅L⋅Δt, where L is cell length, α is growth rate and Δt is the discretised timestep (Figure 2). Growth is constrained by forces imposed by intercellular contacts and via viscous drag resulting in reduced growth within the center of the colony and increased growth at the colony edge (Figure 2) [57, 79]. A cell’s target length is chosen from a normal distribution based on our own microscopy cell length measurements. Once the cell reaches its target length it divides in half creating two daughter cells. Upon birth the orientations of the daughter cells are slightly perturbed simulating imperfections in cell shape leading to buckling of cell files [79]. Cells are simulated as non-motile and have no aggregating properties, cell movement therefore was only caused by growth and cell-to-cell forces. The simulations presented here are constrained to two-dimensions, simulations conducted within three dimensions did not produce significantly different results from the two-dimensional model (data not shown). Moreover, microscopy of colony populations indicated that the vertical structure remained constant throughout the *z-*dimension of the colonies (Figures S4B and S4C).

The model simulates the interaction between three cell types: targets, inhibitors and inhibited targets (Figure 2A). Sustained contact between a target and inhibitor cell will lead to the inhibition of the target cell, reducing its growth rate by a set percentage (δ). The contact time required before inhibition is determined by the inhibition rate (η) and calculated upon initial contact between targets and inhibitors by an integrated Gibson-Bruck Next Reaction Method [56]. The algorithm is based on the same mathematical assumptions as the Gillespie algorithm [80] which generates a time trajectory for all reactions within the stochastic system based on the propensity for each given reaction. If contact between target and inhibitor is lost before inhibition occurs the reaction is removed from the system. If an inhibited target cell loses contact with an inhibitor, the target cells can revert back to their original uninhibited growth rate with the recovery rate (μ) controlled by the Next Reaction Method. The recovery rate was maintained at a constant low level for all simulations [51, 65], however simulations indicated that altering recovery rate had little impact on the outcome of the simulations due to a low probability of cells losing contact within the timescale of the simulations.

Simulations were inoculated randomly with equal ratios of inhibitors to targets within a central circle with a radius of 200 μm (Figure 2C) at three different densities, 1.6, 16 and 160 cells/1000 μm^2^. Within the models examining the cost of CDI carriage, the growth rate of the inhibitor cells was reduced by 5 or 10% relative to the target cells, otherwise both strains had the same unconstrained growth rate. Cells were then allowed to grow, interact and compete for space replicating the growth of a mixed colony growing on a solid surface. Simulations were run for 700 timesteps (equating to simulated 35 h) resulting in colonies of approximately 150,000 cells with an area of 4 × 10^5^ μm^2^. Ten replicates of each parameter set were performed on Nvidia 980 graphics cards. A full list of parameters and notation used within the model are presented in Table S3.

#### Sector frequency and sector size analysis

The colonies produced by the simulations where split into annuli of width 20 μm, and experimental data were split into annuli with a width of 50 pixels (0.225 mm). The maximum size of the simulated colonies was approximately a factor of 10 smaller than the experimental colonies due to limitations of computational resources. The fraction of inhibitor cells, number of sectors (transitions from inhibitor to target cell type) and sector size were then calculated within each annulus of the radially expanding zone. To calculate the number of sectors and sector size, the position of each cell within each annulus was discretised. The mean cell type at each position across the width of each annulus was calculated using multiple iterations of Gaussian smoothing (with the smoothing kernel sigma set to 3) until smoothing no longer had an effect and all singletons had been removed. The length of each binary strip was then normalized to the radius of largest annulus analyzed. A binary summary of the cell type within each annulus was then used to calculate the sector statistics. The distributions of the patch sizes, as well as the number of sectors, is the underlying relevant quantity of interest and we identify size of largest sector within each annulus as a suitable summary statistic for the resulting truncated distributions.

### Quantification and Statistical Analysis

All statistical analyses were conducted in R (version 3.4.3). Statistical details of experiments and simulations can be found in the figure legends along with exact values of *n* and definitions of center and dispersion. Within the simulations (Figure 3, Figures 4A and 4C, Figure 7 and Figure S3) *n* represents independent replicate runs of the simulation at each parameter set, in colony competition assays (Figures 4D and 4F, Figure 5, and Figure 6) *n* represents independent biological replicate colony competitions. Assumptions of normality were tested using Shapiro-Wilk tests. Significant differences between the neighbor indices were calculated using a one-way ANOVA followed by Tukey’s HSD test to test for multiple comparisons. To test for significant interactions between model parameters based on the endpoint ratios of inhibitor cells a robust Two-way ANOVA for trimmed means was conducted {R package: *WRS2*} as the variance between groups was significantly different as tested by a Levene’s Test for homogeneity of variance {R package: *car*}. To test for significant interactions between model parameters based on sectoring patterns, GLMs were conducted with the number of sector or sector size as the response variables, radius of annulus as a covariate and density, inhibition rate and toxicity as interacting predictors. Differences between endpoint fraction of inhibitor strains within colony competition assays were calculated by Two-way ANOVA with density and CDI system as interacting factors, with Tukey multiple comparison of means. Two-tailed one-sample t tests were used to test if the relative fitness of CDI harboring strains was significantly different from 1, i.e., significantly different from CDI negative resistant MG1655 *bamA*^*STy*^.

### Data and Code Availability

Original data for all figures in the paper is available at FigShare [https://doi.org/10.6084/m9.figshare.9546731.v1]. Simulation code is available at GitHub [https://github.com/mbottery/CDI_cellmodeller]. All raw simulation data has not been deposited in a public repository due to its file size but are available from the Lead Contact.

## Supplemental Information

Document S1. Figures S1–S6 and Tables S1–S3

Document S2. Article plus Supplemental Information
