# Supplementary material for: Spatial Organization of Expanding Bacterial Colonies Is Affected by Contact-Dependent Growth Inhibition
Source: Curr Biol. 2019 Nov 4;29(21):3622–3634.e5. doi: 10.1016/j.cub.2019.08.074 (PMC6839403; doi:10.1016/j.cub.2019.08.074)
Supplement: Document S1. Figures S1–S6 and Tables S1–S3 [file mmc1.pdf]

Current Biology, Volume *29*

Supplemental Information

Spatial Organization of Expanding Bacterial Colonies Is Affected by Contact-Dependent Growth Inhibition

Michael J. Bottery, Ioannis Passaris, Calvin Dytham, A. Jamie Wood, and Marjan W. van der Woude

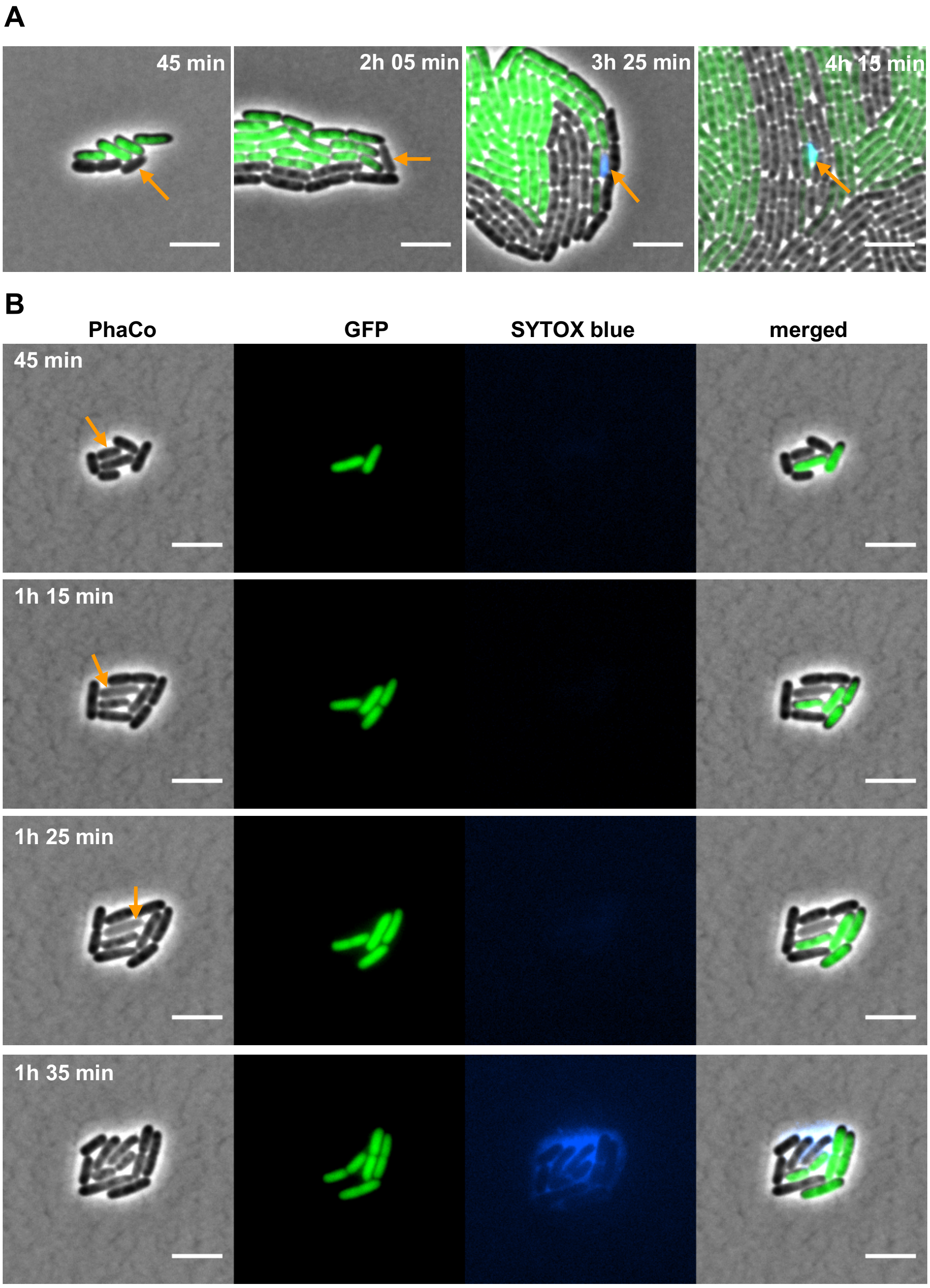

Figure S1. Membrane integrity of target cells is only affected in a small subset of cells when in contact with inhibitor cells (Class I-PFT) in a 5 hours period. Related to Figure 1.

A) Time-lapse fluorescence microscopy images of a single target cell (orange arrows) incorporating the SYTOX blue dye, which can only enter the cell and bind DNA when the membranes are damaged, when in contact with inhibitor cells (GFP positive). Images show the overlay of the phase contrast, GFP and SYTOX blue channels. **B)** Time-lapse microscopy images of a single target cell (orange arrows) showing cell lysis after contact with an inhibitor cell, as indicated by its released chromosomal DNA binding the SYTOX blue dye. Cells were grown on LB agar pads containing 0.5 μM SYTOX blue dye. Scale bars correspond to 5 μm.

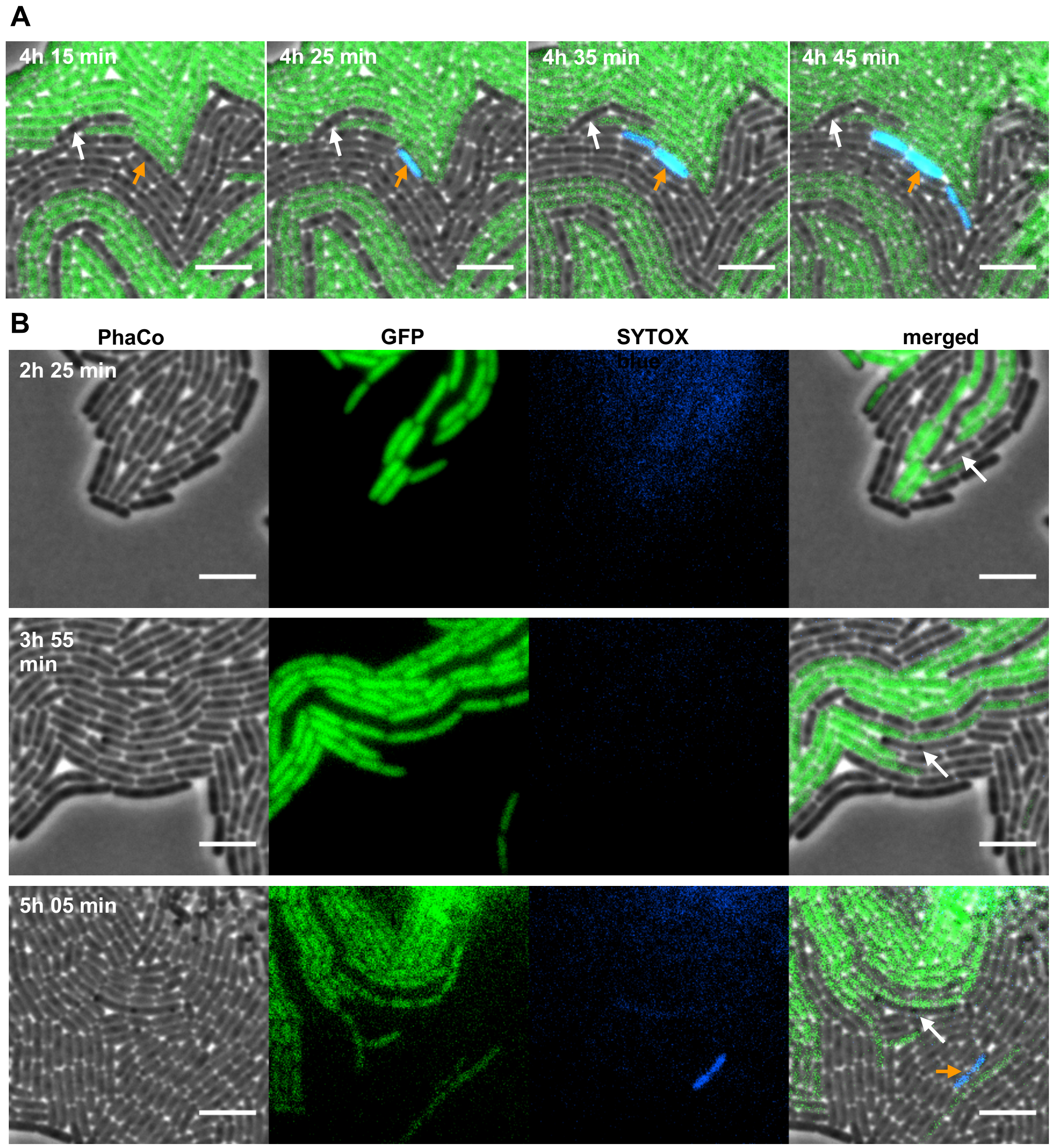

Figure S2. Membrane integrity of target cells is only affected in a small subset of cells when in contact with inhibitor cells (Class II-tRNase) in a 5 hours period. Related to Figure 1.

A) Time-lapse microscopy images off target cells (orange arrows) incorporating the SYTOX blue dye when in contact with inhibitor cells (GFP positive). White arrows indicate a target cell that has been in contact with inhibitor cells during the entire time course, leading to growth arrest but no incorporation off the SYTOX blue dye. Images show the overlay off the phase contrast, GFP and SYTOX blue channels. **B)** Time-lapse microscopy images off a target cell (white arrows) in contact with inhibitor cells, showing growth arrest during the time course off the experiment but no incorporation off the SYTOX blue dye. Orange arrow indicates two target cells that incorporate the SYTOX blue dye at later time stages of the microscopy experiment. Cells were grown on LB agar pads containing 0.5 µM SYTOX blue dye. Scale bars correspond to 5 µm.

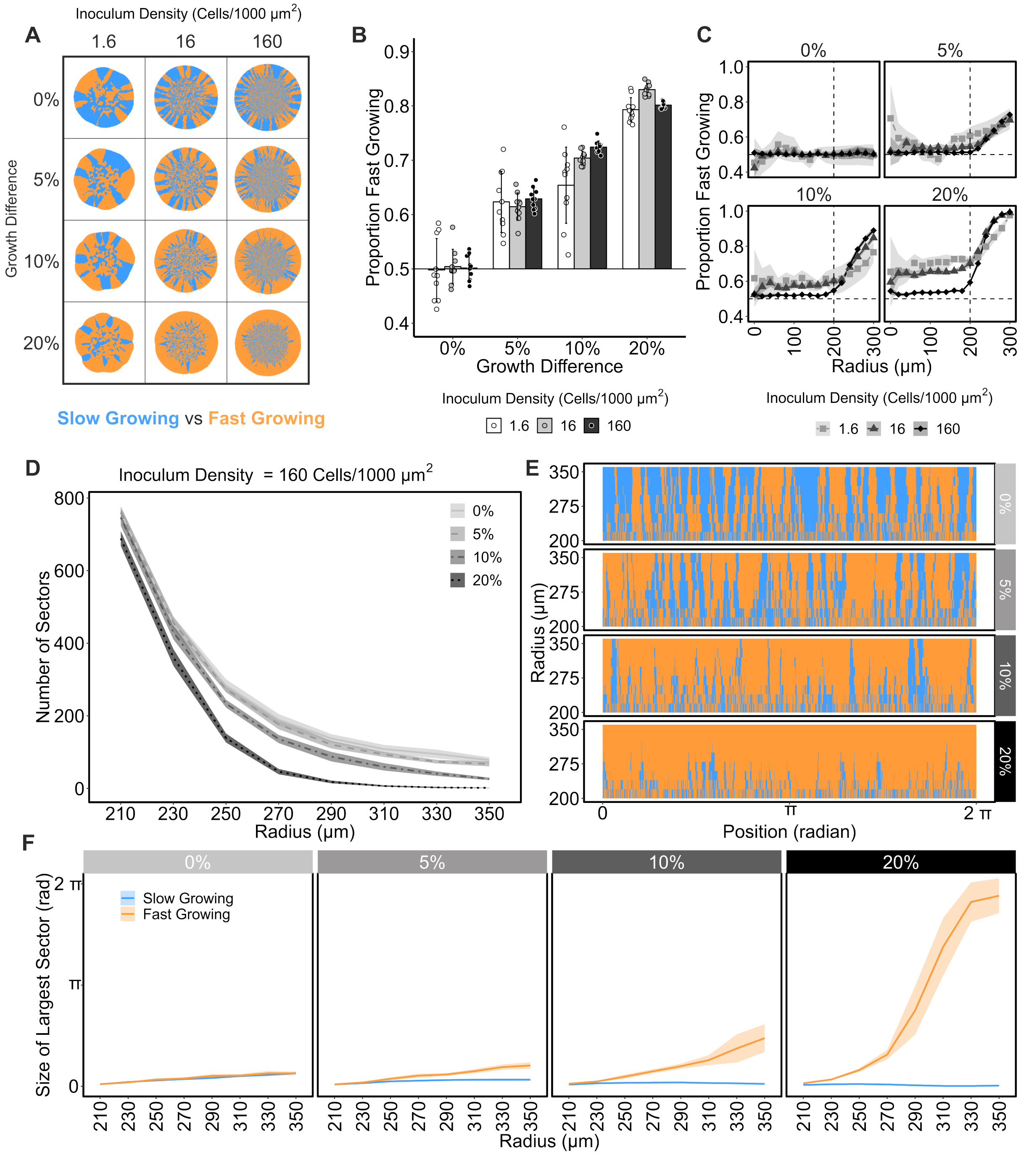

Figure S3. Modelling the effect of growth rate difference and inoculum density on population composition and sectoring. Related to Figure 3 and Figure 4.
A) Representative simulation output of competitions between two non-inhibiting strains with either a 0%, 5%, 10% or 20% growth difference. Blue represents the slower growing strain; orange represents the faster growing strain. Approximately 150,000 cells per simulated colony **B)** Mean end-point proportion of faster growing (orange) strain relative to slower growing (blue) strain. Growth difference had a significant effect on the end-point proportion of faster growing cells, whereas inoculum density did not (Robust two-way ANOVA for trimmed means, density: Q = 1.73, $P = 0.44$, growth difference: Q = 2600.07, $P < 0.01$). Bars shaded by initial inoculum density. Error bars show standard deviation (10 simulations per parameter set). **C)** The mean end point proportion of the faster growing strain at increasing radial annuli. Panel labels show percentage growth difference between fast and slow

growing strains. The horizontal dashed line represents the initial frequency of strains within the simulation and the vertical dashed line represents the radius of the inoculum area. Lines coloured by inoculation cell density and shaded areas represent 95% confidence intervals (n = 10). **D)** The number of total sectors formed in the high-density simulations (160 cells/1000 μm^2) during radial expansion away from the initial inoculum area. Lines coloured by growth difference between competing strains and shaded areas represent 95% confidence intervals (n = 10). **E)** A representation of the normalised strip data used to calculate the number of sectors and size of sectors formed during radial expansion. The colonies were split into annuli with radii between 200 and 350 µm, cell positions within each annulus were normalised to the length of the largest annuli and their position plotted as radians around the colony. **F)** The size of the largest orange and blue sectors in radians across the radially expanding zone. Plot faceted by growth difference between the fast and slow growing strains. Shaded areas represent 95% confidence intervals (n = 10).

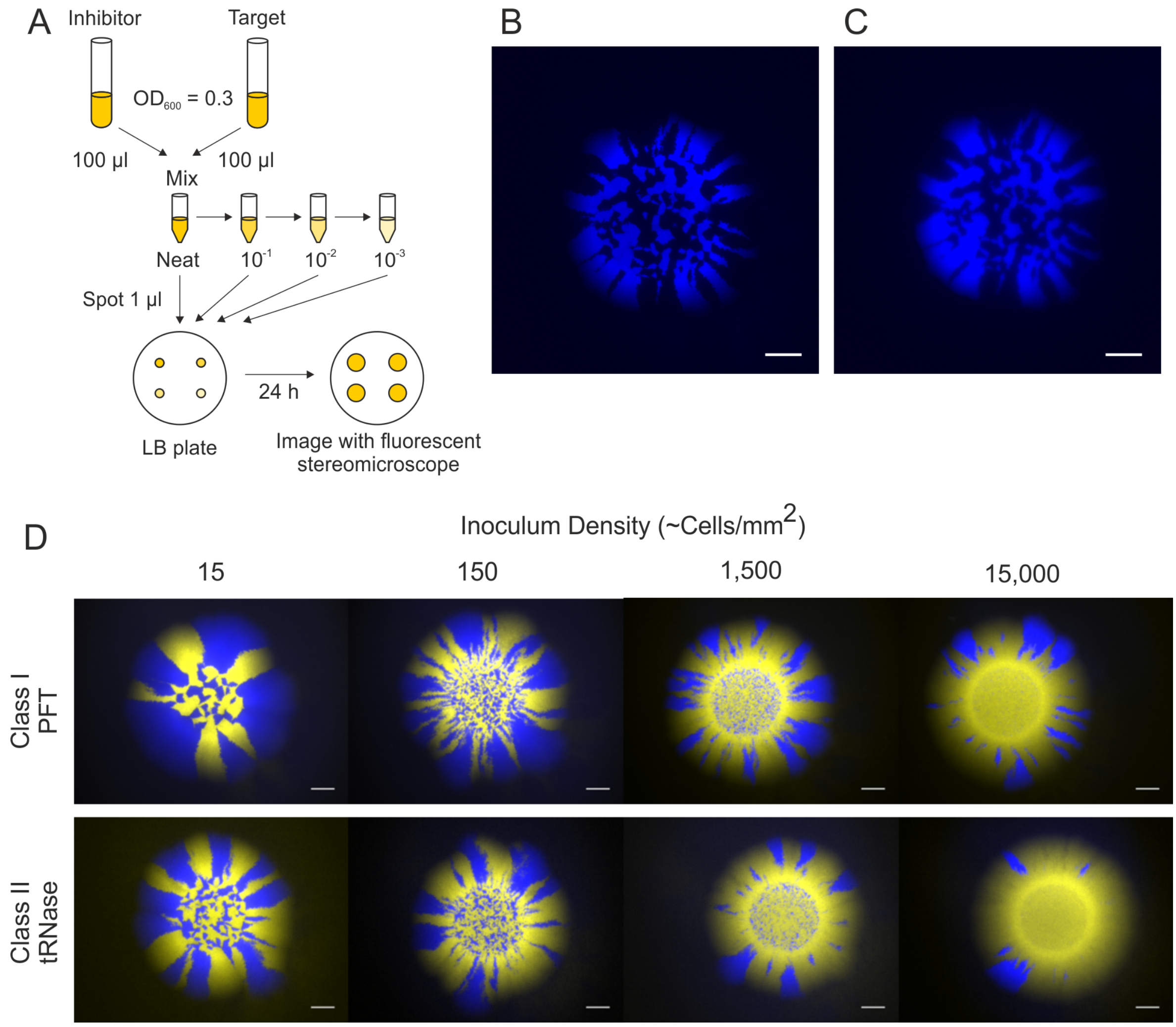

Figure S4. Colony competition experiments. Related to Figure 5.
A) Target and inhibitor cells expressing GFP or mCherry, respectively, were grown to mid-exponential phase and then mixed at a 1:1 ratio before serially diluting to 10^{-3}. The ratio and density of the mixtures were confirmed through plating for single cells on LB and strains were distinguished by their fluorophores. 1 µl of the mixture at each dilution was spotted onto a LB agar plate. The initial inoculum spot had a radius of approximately 1.5 mm. Colonies were imaged using a fluorescent stereomicroscope following 24 hours of competition at 37˚C and ratios of targets and inhibitors were assessed through image analysis. **B/C)** Two strain colony structure is maintained throughout the z-plane. A mixed colony containing two differential marked strains of *E. coli* were spotted onto LB agar and allowed to complete for 24 hours. Images of the colony after 24 hours from **B)** the top of the colony and **C)** the bottom of the colony show the same patterning, thus the vertical spatial patterning is maintained throughout the z-plane of the colonies. **D)** Swapping fluorophore has no effect on the outcome of competition. Representative fluorescent stereomicroscope images of colony competitions between target and inhibitor cells with increasing inoculum density. The target strain expresses GFP and is false coloured blue, while the No-Toxin, Class I-PFT and Class II-tRNAse strains express mCherry and are false coloured yellow. Scale bars correspond to 1 mm. Images are representative of 6 replicate competitions, 3 with the CDI system in the GFP background and 3 with the CDI system in the mCherry background.

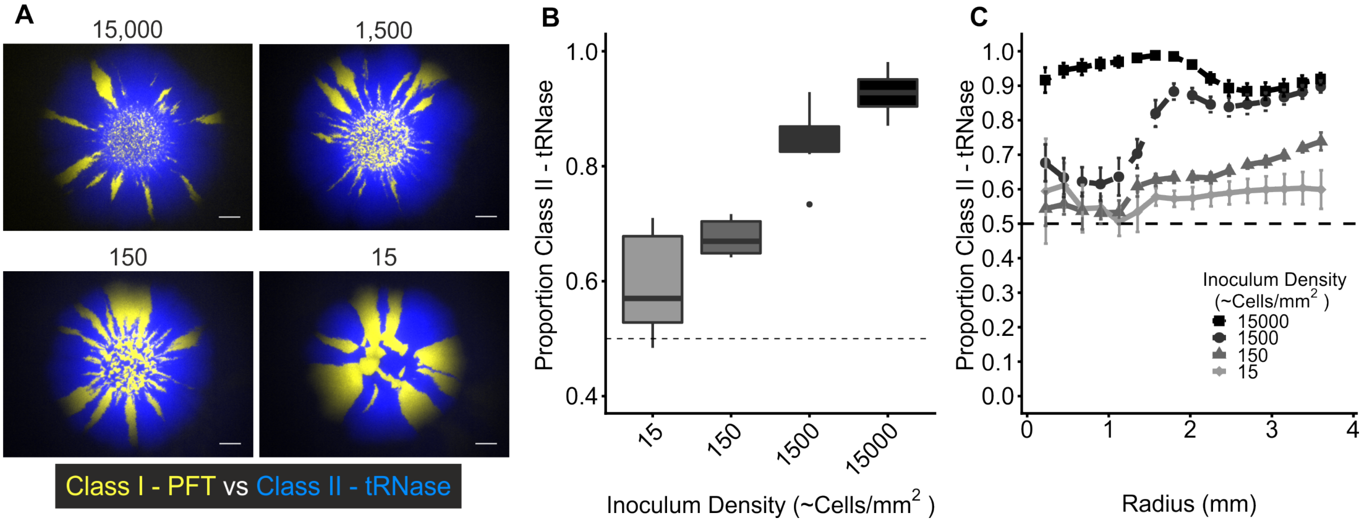

Figure S5 ***E. coli*** **expressing Class II-tRNase CDI system inhibits the growth of** ***E. coli*** **expressing the Class I-PFT CDI system. Related to Figure 5.**

A) Fluorescent stereomicroscope images of colony competitions between Class I-PFT expressing (mCherry, false coloured yellow) and Class II-expressing (GFP, false coloured blue) cells with increasing inoculum density. Scale bars correspond to 1 mm. Images are representative of 6 replicate competitions (3 with the Class I-PFT system in the GFP background and 3 with the Class I-PFT system in the mCherry background). **B)** The proportion of Class II-tRNase expressing cells as determined by fluorescence within colonies after 24 hours of competition. Boxplots show median, $25^{th}/75^{th}$ percentiles, 1.5 * IQR and outliers (n = 6). **C)** The proportion of Class II-tRNase expressing cells plotted against increasing radial annuli after 24 hours of competition within colonies. Error bars represent SEM (n = 6). **B/C** dashed horizontal line represents initial ratio of inhibitor cells within the inoculum.

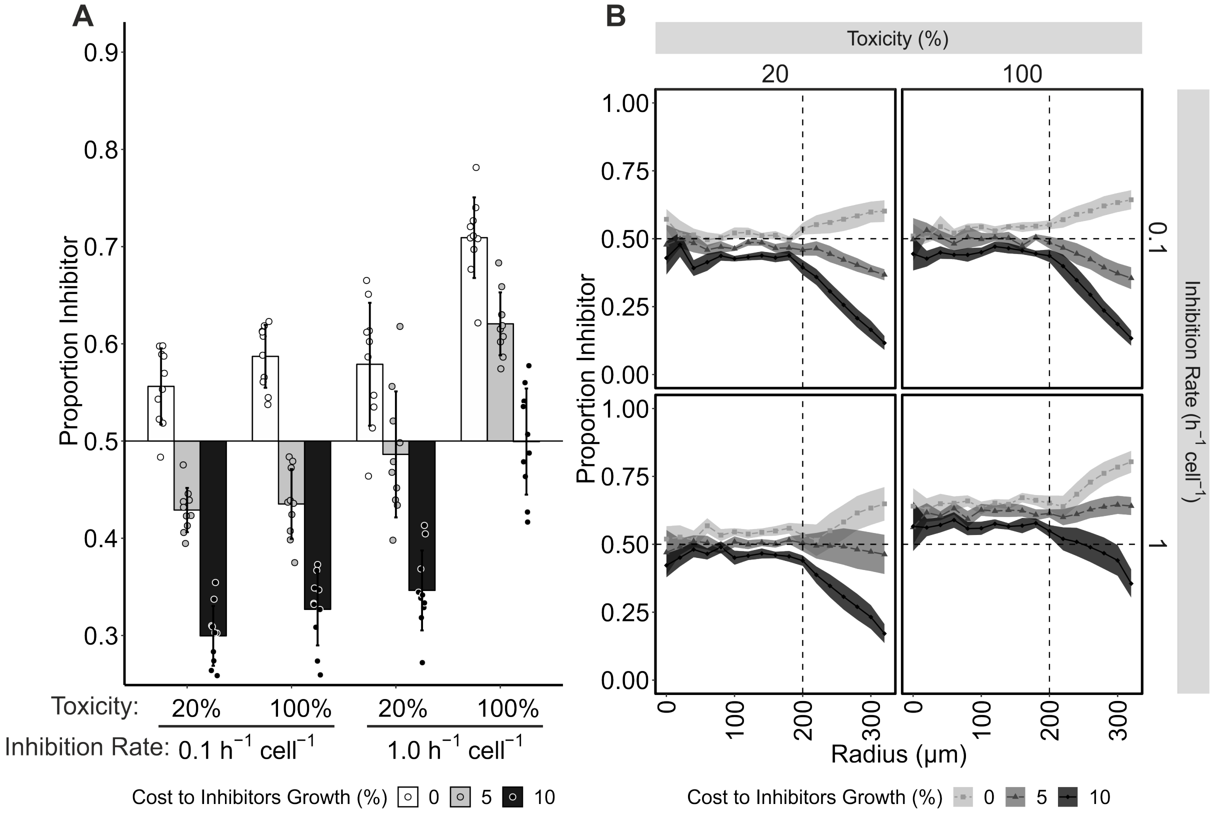

Figure S6. Computational modelling predicts that costs imposed upon inhibitors by CDI systems can outweigh the benefit when the potency of CDI is low. Related to Figure 7.

A cost to the inhibitor cells was imposed as a 0%, 5% or 10% reduction in growth rate and simulations were then run with the same parameters as simulations presented in Figure 3. Simulation results of low cell-density inoculum (16 cells/1000 µm^2) **A)** The mean end-point proportion of inhibitor cells (I) relative to target cells (T) following simulated competition with target cells. Bars coloured by the level of cost imposed by the CDI system upon the inhibiting cells. Error bars represent SEM (10 simulations per parameter set.) **B)** The mean proportion of inhibitor cells at increasing radial annuli after simulated competition with target cells. Horizontal dashed line represents initial inoculum ratio of inhibitors to targets, vertical dashed line represents initial inoculum radius. Shaded areas represent 95% confidence intervals (n = 10).

Primer name	Sequence (5'-3')	Purpose
oMV939	TTGCTCCGGGCTATGAAATAGAAAAATGAATCCGTTGAAGACTAGTGCTTGGATTCTCACCA	Construction of *E. coli* MG1655 *attB::Km-gfp* (MV1463) with pZE21-gfp as DNA template for PCR and *E. coli* MG1655::*attB-Km-mCherry* (MV1488) with pMV371 as DNA template; Fw primer
oMV940	ATTAAAAACAACTTTTTGTCTTTTTACCTTCCCGTTTCGCCCTAGGTCTAGGGCGGCGGATTTGT	Construction of *E. coli* MG1655 *attB::Km-gfp* (MV1463) with pZE21-gfp as DNA template for PCR and *E. coli* MG1655::*attB-Km-mCherry* (MV1488) with pMV371 as DNA template; Rev primer
oMV1367	CAGTGAATTCAGCGGTGGCGGTGGCGTGAGCAAGGGCGAGGCAGT	Amplification of *mScarlet* gene from pmScarlet-C1; contains a 5' EcoRI RE site; Fw primer
oMV1368	TGCTCCCGGGTTAACTTGTACAGCTCGTCCATG	Amplification of *mScarlet* gene from pmScarlet-C1; contains a 5' XmaI RE site; Rev primer
oMV1373	GCTCGAATTCACTGGCCGTC	Amplification of pDHL1029 backbone from pDHL1029-msfGFP; contains a 5' EcoRI RE site; Fw primer
oMV1374	ATAACCCGGGGTGTAGGCT	Amplification of pDHL1029 backbone from pDHL1029-msfGFP; contains a 5' XmaI RE site; Rev primer
oMV1289	CTTTAAAAATAGGAATCCCCATAGGCTTTATTCTTGGGCTGGGTTTGTGGGGCCTTAGTATCTTAGACAGAAAATAGATTAAAGAGGAGAAATTAAGCATGAGTAAAGGTGAAGAACTGT	Construction of pCC1-*cdiBAI*^{EC93}-*msfGFP*. Amplification of *msfGFP-frt-Km-frt* from pDHL1029-*msfGFP*. Contains the ribosome binding site from the phage λ CI protein; Fw primer
oMV1290	ACCCCAATTTCCAGTAACTGTTCAGCAACCTTTGTCCGACATGGAGCAGCAACCGCACAGCCTCTTGCTATCAAGCTCAGCGAGTTATTCTCAACAACAAATTCCGGGGATCCGTCGACC	Construction of pCC1-*cdiBAI*^{EC93}-*msfGFP*, pCC1-*cdiBAI*^{EC93}-*mScarlet*, pCC1-*ΔcdiA-CT-ΔcdiI*^{EC93}-*msfGFP* and pCC1-*ΔcdiA-CT-ΔcdiI*^{EC93}-*mScarlet*. Amplification of *msfGFP-frt-Km-frt* or *mScarlet-frt-Km-frt* from, respectively, pDHL1029-msfGFP or pMV528; Rev primer
oMV1500	CTTTAAAAATAGGAATCCCCATAGGCTTTATTCTTGGGCTGGGTTTGTGGGGCCTTAGTATCTTAGACAGAAAATAGATTAAAGAGGAGAAATTAAGCATGGTGAGCAAGGGCGAGGCA	Construction of pCC1-*cdiBAI*^{EC93}-*mScarlet*. Amplification of *mScarlet-frt-Km-frt* from pMV528. Contains the ribosome binding site from the phage λ CI protein; Fw primer
oMV1279	GAGTGAACAGGAACGTCAGC	Diagnostic Fw primer for pCC1-*cdiBAI*^{EC93}-*msfGFP* and pCC1-*ΔcdiA-CT-ΔcdiI*^{EC93}-*msfGFP*
oMV1291	ATGATCCAGTTCATCAGCGGTC	Diagnostic Rev primer for pCC1-*cdiBAI*^{EC93}-*msfGFP* and pCC1-*ΔcdiA-CT-ΔcdiI*^{EC93}-*msfGFP*
oMV1316	GCTAGAACCGTCAGATATTAACGATGATTTAAGAAAAGATATATTAAAAATCAATCAGATAATTGTATAAATTAAAGAGGAGAAATTAAGCATGAGTAAAGGTGAAGAACTGT	Construction of pCC1-*cdiBAI*^{UPEC536}-*msfGFP*. Amplification of *msfGFP-frt-Km-frt* from pDHL1029-*msfGFP*. Contains the ribosome binding site from the phage λ CI protein; Fw primer
oMV1317	GACTTTCACCCCCAAGCTGCTGAACATGCCCGGCACACAAAGAAGATCTCGGCTCAGTGGCCGGGATTAGATTCCGGGGATCCGTCGACC	Construction of pCC1-*cdiBAI*^{UPEC536}-*msfGFP*, pCC1-*cdiBAI*^{UPEC536}-*mScarlet*, pCC1-*ΔcdiA-CT-ΔcdiI*^{UPEC536}-*msfGFP* and pCC1-*ΔcdiA-CT-ΔcdiI*^{UPEC536}-*mScarlet*. Amplification of *msfGFP-frt-Km-frt* or *mScarlet-frt-Km-frt* from, respectively, pDHL1029-*msfGFP* or pMV528; Rev primer
oMV1501	GCTAGAACCGTCAGATATTAACGATGATTTAAGAAAAGATATATTAAAAATCAATCAGATAATTGTATAAATTAAAGAGGAGAAATTAAGCATGGTGAGCAAGGGCGAGGCA	Construction of pCC1-*cdiBAI*^{UPEC536}-*mScarlet*. Amplification of *mScarlet-frt-Km-frt* from pMV528. Contains the ribosome binding site from the phage λ CI protein; Fw primer
oMV1488	GGCTTGTCGGCAACAGTACAAGTGCTGCCGGTACGGGGGCACAGGCAGGTAGGAACTCGGTTGAGAATAATGCATGAATTAAAGAGGAGAAATTAAGCATGAGTAAAGGTGAAGAACTGT	Construction of pCC1-*ΔcdiA-CT-ΔcdiI*^{EC93}-*msfGFP*. Amplification of *msfGFP-frt-Km-frt* from pDHL1029-*msfGFP*. Contains the ribosome binding site from the phage λ CI protein; Fw primer

Primer name	Sequence (5'-3')	Purpose
oMV1489	GCATTGCCAGTGGCGATGTGGCTGGCGCGGCTGCTGGAGCTGGTGCCGGGAAGAACGTTGTTGAGAATAATGCGTGAATTAAAGAGGAGAAATTAAGCATGAGTAAAGGTGAAGAACTGT	Construction of pCC1-*ΔcdiA-CT-ΔcdiI*^{UPEC536}-*msfGFP*. Amplification of *msfGFP-frt-Km-frt* from pDHL1029-*msfGFP*. Contains the ribosome binding site from the phage λ CI protein; Fw primer
oMV1498	GGCTTGTCGGCAACAGTACAAGTGCTGCCGGTACGGGGGCACAGGCAGGTAGGAACTCGGTTGAGAATAATGCATGAATTAAAGAGGAGAAATTAAGCATGGTGAGCAAGGGCGAGGCA	Construction of pCC1-*ΔcdiA-CT-ΔcdiI*^{EC93}-*mScarlet*. Amplification of *mScarlet-frt-Km-frt* from pMV528. Contains the ribosome binding site from the phage λ CI protein; Fw primer
oMV1499	GCATTGCCAGTGGCGATGTGGCTGGCGCGGCTGCTGGAGCTGGTGCCGGGAAGAACGTTGTTGAGAATAATGCGTGAATTAAAGAGGAGAAATTAAGCATGGTGAGCAAGGGCGAGGCA	Construction of pCC1-*ΔcdiA-CT-ΔcdiI*^{UPEC536}-*mScarlet*. Amplification of *mScarlet-frt-Km-frt* from pMV528. Contains the ribosome binding site from the phage λ CI protein; Fw primer
oMV1324	ACTGACAGAAGATCAGAAGC	Diagnostic Fw primer for pCC1-*cdiBAI*^{UPEC536}-*msfGFP* and pCC1-*ΔcdiA-CT-ΔcdiI*^{UPEC536}-*msfGFP*
oMV1325	CATCGTTCGCATAGTACGTC	Diagnostic Rev primer for pCC1-*cdiBAI*^{UPEC536}-*msfGFP* and pCC1-*ΔcdiA-CT-ΔcdiI*^{UPEC536}-*msfGFP*
oMV1346	AGGTGAGACTATAGAATACTCAAGCTTGCATGCCTGCAGGTCGACTCTAGAGGATCCCACATTAAAGAGGAGAAATTAAGCATGAGTAAAGGTGAAGAACTGT	Construction of pCC1-*msfGFP*. Amplification of *msfGFP-frt-Km-frt* from pDHL1029-*msfGFP*; Fw primer
oMV1347	GACGTTGTAAAACGACGGCCAGTGAATTGTAATACGACTCACTATAGGGCGAATTCGAGCTCGGTACCCGGGGATCCCACATTCCGGGGATCCGTCGACC	Construction of pCC1-*msfGFP* and pCC1-*mScarlet*. Amplification of *msfGFP-frt-Km-frt* or *mScarlet-frt-Km-frt* from, respectively, pDHL1029-*msfGFP* or pMV528; Rev primer
oMV1490	AGGTGAGACTATAGAATACTCAAGCTTGCATGCCTGCAGGTCGACTCTAGAGGATCCCACATTAAAGAGGAGAAATTAAGCATGGTGAGCAAGGGCGAGGCAGT	Construction of pCC1-*mScarlet*. Amplification of *mScarlet-frt-Km-frt* from pMV528; Fw primer
oMV1294	CAGGGTCAGTTTACCGTTGG	Diagnostic Fw primer for pCC1-*msfGFP*
oMV1359	TTATACGCAAGGCGACAAGG	Diagnostic Rev primer for pCC1-*msfGFP* and pCC1-*mScarlet*
oMV1457	TCGATCTCGAACTCGTGGC	Diagnostic Fw primer for pCC1-*mScarlet*
oMV1521	ATGGAGACAAAGCCGAGCAGTTCCAGTTTAACATTGGTAAAACCTGGTAAATTCCGGGGATCCGTCGACC	Construction of *S*. Typhimurium *bamA-frt-Km-frt*. Amplification of *frt-Km-frt* from pDHL1029-*msfGFP*; Fw primer
oMV1522	TCGCCAAAAGTCATCGCTACACTACCAATGCATTCCTTTGCAGTGAACAAGTGTAGGCTGGAGCTGCTTC	Construction of *S*. Typhimurium *bamA-frt-Km-frt*. Amplification of *frt-Km-frt* from pDHL1029-*msfGFP*; Rev primer
oMV1522	TCTATAACGACTTCCAGGCG	Diagnostic Fw primer for *S*. Typhimurium *bamA-frt-Km-frt*.
oMV1402	CCTTACCATCCCATTTGCAC	Diagnostic Rev primer for *S*. Typhimurium *bamA-frt-Km-frt*.
oMV1526	TATCCGAGCGGGTTCAAGACTTTTGTTATCGCATTGGCTCGATTCTGCTGGTGCTGTTAATGGGGCTTGCACTTTTCAATGATTTCTCTCGGTTATGAGAGAGTTAGTTAGGAAGAACGC	Construction of *E. coli* MG1655 *bamA*^{STy}-*frt-Km-frt*. Amplification of *bamA*^{STy}-*frt-Km-frt* from genomic DNA of *S*. Typhimurium *bamA-frt-Km-frt*; Fw primer
oMV1527	TTTGCACCGGAGGGTGCAGTTCTTTGCGTGGCCCGGCGATCTTATATTGATCGCCTAAAGTCATCGCTACACTACCACTACATTCCTTTGTGGAGAACACGTGTAGGCTGGAGCTGCTTC	Construction of *E. coli* MG1655 *bamA*^{STy}-*frt-Km-frt*. Amplification of *bamA*^{STy}-*frt-Km-frt* from genomic DNA of *S*. Typhimurium *bamA-frt-Km-frt*; Rev primer

Table S1. Primers used throughout this study. Related to Figures 1, 4, 5 and 6 and STAR Methods.

Plasmids-See Table S2		
pKD46	[S1]	
pDHL1029-*msfGFP*	[S2]	
pZE21-*gfp*	[S3]	
pmCherry	Clontech	Cat. No. 632522
pZE21-*mCherry* (pMV371)	King A. (PhD thesis, U of York)	N/A
pmScarlet-C1	Bindels et al https://www.nature.com/articles/nmeth.4074	N/A
pDHL1029-*mScarlet* (pMV528)	This paper	N/A
pCP20	[S4]	N/A
pCC1FOS	Lucigen (Epicentre) Accession EU140751	CCFOS110
pCC1FOS-*msfGFP* (pMV531)	This paper	N/A
pCC1FOS-*mScarlet* (pMV532)	This paper	N/A
pCC1FOS-*cdiBAI*^{EC93}-*msfGFP* (pMV476)	This paper and [S5]	N/A
pCC1FOS-*cdiBAI*^{EC93}-*mScarlet* (pMV533)	This paper and [S5]	N/A
pCC1FOS-*cdiBAI*^{UPEC536}-*msfGFP* (pMV485)	This paper and [S5]	N/A
pCC1FOS-*cdiBAI*^{UPEC536}-*mScarlet* (pMV534)	This paper and [S5]	N/A
pCC1FOS-*ΔcdiA-CT-ΔcdiI*^{EC93}-*msfGFP* (pMV535)	This paper and [S5]	N/A
pCC1FOS-*ΔcdiA-CT-ΔcdiI*^{EC93}-*mScarlet* (pMV536)	This paper and [S5]	N/A
pCC1FOS-*ΔcdiA-CT-ΔcdiI*^{UPEC536}-*msfGFP* (pMV537)	This paper and [S5]	N/A
pCC1FOS-*ΔcdiA-CT-ΔcdiI*^{UPEC536}-*mScarlet* (pMV538)	This paper and [S5]	N/A
pBR322	[S6]	N/A
pDAL776	This paper and [S5]	N/A

Table S2. Plasmids used throughout this study. Related to Figures 1, 4, 5, 6, 7 and STAR Methods

Symbol	Parameter	Units	Value	Source
**Biophysics**				
Δt	Time step	h	0.05	
γ	Cell growth drag	-	10	[S7]
N_{cont}	Max contacts	-	24	[S7]
N_{sub}	Number of sub-steps	-	8	[S7]
ε_{div}	Division orientation noise	%	0.1	[S7]
**Cell States**				
r	Cell radius	μm	0.5	[S7]
L_{target}	Division length	μm	3.5	This study
σ	Division standard deviation	μm	0.5	This study
α	Unconstrained Growth rate	h^{-1}	1	This study
β	Cost to inhibitors due to CDI expression	%	0, 5, 10	This study
δ	Growth reduction of inhibited target	%	20, 100	This study
η	Inhibition rate	$Cell^{-1}$ h^{-1}	0.1, 1.0	This study
μ	Recovery rate	$Cell^{-1}$ h^{-1}	0.1	This study
**Initiation**				
r_{init}	Inoculation radius	μm	200	This study
D_{init}	Inoculation density	Cell/1000 μm^2	16, 160, 1600, 16000	This study
$I:T$	Inhibitor to target ratio	-	1:1	This study

Table S3. Parameter notation and values used within the individual based modelling. Related to Figure 2, 3, 4 and 7.

Supplemental References

S1. Datsenko, K. A. & Wanner, B. L. One-step inactivation of chromosomal genes in Escherichia coli K-12 using PCR products. *Proc Natl Acad Sci U S A* **97,** 6640–6645 (2000).

S2. Ke, N., Landgraf, D., Paulsson, J. & Berkmen, M. Visualization of Periplasmic and Cytoplasmic Proteins with a Self-Labeling Protein Tag. *Journal of Bacteriology* **198,** 1035–1043 (2016).

S3. Da Re, S., Quéré, B. L., Ghigo, J.-M. & Beloin, C. Tight Modulation of Escherichia coli Bacterial Biofilm Formation through Controlled Expression of Adhesion Factors. *Appl. Environ. Microbiol.* **73,** 3391–3403 (2007).

S4. Cherepanov, P. P. & Wackernagel, W. Gene disruption in Escherichia coli: TcR and KmR cassettes with the option of Flp-catalyzed excision of the antibiotic-resistance determinant. *Gene* **158,** 9–14 (1995).

S5. Aoki, S. K. *et al.* A widespread family of polymorphic contact-dependent toxin delivery systems in bacteria. *Nature* **468,** 439–442 (2010).

S6. Bolivar, F., Rodriguez, R.L., Greene, P.J., Betlach, M.C., Heyneker, H.L., Boyer, H.W., Crosa, J.H., and Falkow, S. (1977). Construction and characterization of new cloning vehicle. II. A multipurpose cloning system. Gene 2, 95–113.

S7. Rudge, T. J., Steiner, P. J., Phillips, A. & Haseloff, J. Computational Modeling of Synthetic Microbial Biofilms. *ACS Synth. Biol.* **1,** 345–352 (2012).
